# Polymeric Systems for the Controlled Release of Flavonoids

**DOI:** 10.3390/pharmaceutics15020628

**Published:** 2023-02-13

**Authors:** Gianni Pecorini, Elisabetta Ferraro, Dario Puppi

**Affiliations:** 1 BIOLab Research Group, Department of Chemistry and Industrial Chemistry, University of Pisa, UdR INSTM Pisa, Via Moruzzi 13, 56124 Pisa, Italy; 2 Department of Biology, University of Pisa, S.S. 12 Abetone e Brennero 4, 56127 Pisa, Italy

**Keywords:** polyphenolic compounds, phytotherapeutics, bioavailability, drug delivery, natural polymers, synthetic polymers, biomedical devices, nanoparticles, scaffolds

## Abstract

Flavonoids are natural compounds that are attracting great interest in the biomedical field thanks to the wide spectrum of their biological properties. Their employment as anticancer, anti-inflammatory, and antidiabetic drugs, as well as for many other pharmacological applications, is extensively investigated. One of the most successful ways to increase their therapeutic efficacy is to encapsulate them into a polymeric matrix in order to control their concentration in the physiological fluids for a prolonged time. The aim of this article is to provide an updated overview of scientific literature on the polymeric systems developed so far for the controlled release of flavonoids. The different classes of flavonoids are described together with the polymers most commonly employed for drug delivery applications. Representative drug delivery systems are discussed, highlighting the most common techniques for their preparation. The flavonoids investigated for polymer system encapsulation are then presented with their main source of extraction and biological properties. Relevant literature on their employment in this context is reviewed in relationship to the targeted pharmacological and biomedical applications.

## 1. Introduction

Flavonoids represent a class of natural products produced by plants as secondary metabolites [1] comprising more than 6000 different structures [2]. Most of them are colored substances (flavonoid from the Latin “*flavus,*” meaning yellow), and thus they are the source of color of leaves, flowers, and fruits of many plants along with other molecules, such as carotenoids and chlorophyll [2]. Plant flavonoids present many biological activities since they can act as signaling molecules, UV filters, and reactive oxygen species (ROS) scavengers. Moreover, they present several functional activities in drought, heat, and freezing tolerance [3], as well as in protecting plants from pathogen infection and insect feeding [4]. The biosynthesis mechanism of flavonoids in plants starts from phenylalanine, and it follows the phenylpropanoid pathway [5]. The first step involves the transformation of phenylalanine into 4-coumaroyl-CoA, which enters the flavonoid biosynthesis pathway [6]. The chalcone synthase is the first enzyme specific for the flavonoid pathway, and its role is to produce the chalcone scaffolds from which all flavonoids derive. The obtained flavonoids accumulate in vacuoles of plant cells in the form of glycosides [7]. Flavonoids induce several health benefits in humans thanks to their antioxidant, anti-inflammatory, anticancer, cardioprotective, antimicrobial, and antiviral properties [3]. For these reasons, they have been extensively studied for application in cosmetic products, as well as for biomedical and pharmacological applications. Dermatology and cosmetics are among the most common applications of flavonoids [8], for example, in the formulation of sunscreens. This is mostly due to their antiradical properties, which support their ability to absorb ultraviolet radiation in the range 250–280 nm (UVB) and 350–385 nm (UVA) [8,9].

Flavonoid encapsulation into a polymeric matrix has been extensively investigated as an effective means for their targeted and controlled release, as well as for their protection from oxidation and decomposition, which have been demonstrated to occur in aqueous-oxygenated mediums at physiological pHs [7,10,11]. Topical administration is a widely investigated field of application of encapsulated flavonoids aimed at exploiting the bioactivity of these compounds and more effectively penetrating the skin layers [12]. In addition, implantable devices made of biodegradable polymers and able to release flavonoids in situ have recently been investigated for guided tissue regeneration, as well as to obtain high local concentrations of the bioactive agents for prolonged periods [8,13,14].

This review article is aimed at providing an updated overview of the current state of the art on flavonoid-loaded polymeric systems. In particular, flavonoids are first introduced by highlighting their chemical-physical properties, bioactivity, and bioavailability. Then, the polymeric systems proposed for improving the controlled release of each flavonoid are discussed.

## 2. Flavonoids

The chemical structure of flavonoids is generally characterized by the presence of three rings named A, B, and C. A and B are aromatic rings, while C is a pyran ring. The A ring is fused with the C ring, and the B ring is covalently bonded to the C ring. Depending on their structural differences, in particular on the oxidation degree of the C ring, flavonoids can be divided into seven different subclasses: flavanols, flavones, isoflavones, anthocyanidins, flavanones, flavonols, and chalcones [15].

**Figure 1:**
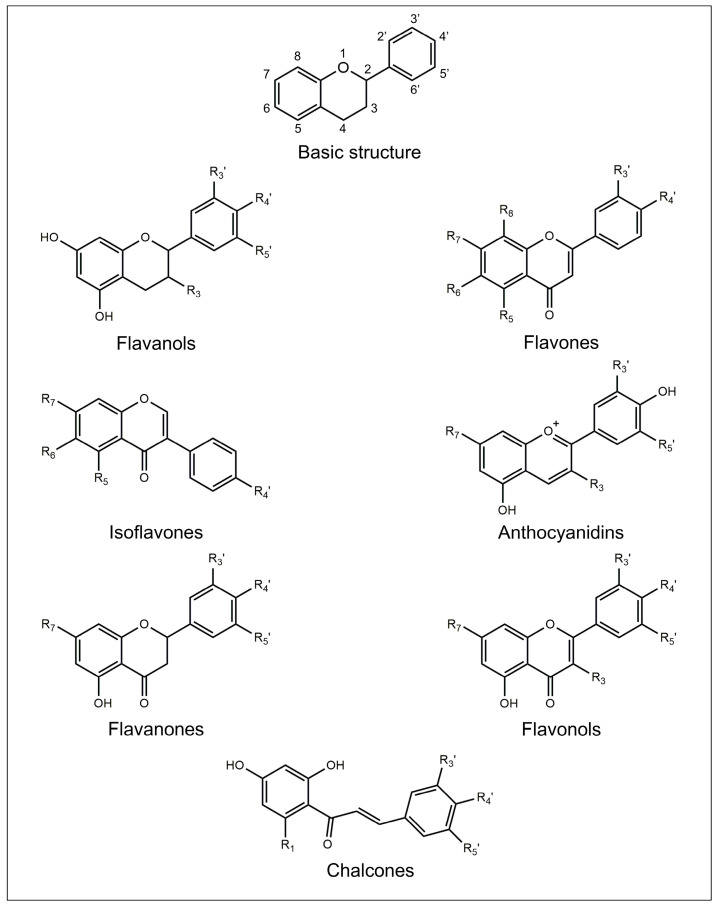
Basic chemical structure of flavonoids and general molecular structures of the different flavonoid subclasses.

The chemical structures of the main flavonoid subclasses are depicted in Figure 1.

### 2.1. Flavanols

Flavanols, also known as flavan-3-ols, are characterized by a C pyran ring and a hydroxyl group bonded to the carbon in the C3 position [16]. Flavanols are usually found as aglycones, and they also exist in the form of oligomers and polymers. The monomers are called catechins and epicatechins, while the oligomers and polymers are called proanthocyanidins or condensed tannins. The monomers are differentiated by the stereochemistry of two asymmetric carbons C2 and C3, the presence of galloyl groups, as well as the level of hydroxylation of ring B [16,17]. They are naturally found in wood, bark, cereals, seeds, fruits (e.g., apples, grapes, pears, apricots, and blueberries), and beverages (e.g., chocolate, red wine, cider, tea, and beer) [16].

### 2.2. Flavones

Flavones are characterized by the presence of a double bond between C2 and C3 in the C ring, the absence of substitution at the C3 position, and a carbonyl group at the C4 position [18]. Together with flavonols, they are the primary pigments in white and cream-colored flowers, and they act as co-pigments with anthocyanins in blue flowers. They are generally found in plants as 7-*O*-glycosides, 6-*C*-glucosides, and 8-*C*-glucosides and may also have acetyl or malonyl moieties. They are commonly found in chamomile flowers, plants from the mint family, grapefruit, juice from fruits belonging to the citrus family (e.g., bergamot, mandarin orange, orange, and citron fruits), wine, olive oil, honey, vegetables (e.g., sunflower family, carrot family, parsley, chicory), cereals, and legumes (e.g., millet, sorghum) [18].

### 2.3. Isoflavones

The general structure of isoflavones is characterized by the B ring bonded to the C ring in the C2 position [19]. Their main role is to affect plant-microbe interactions. They show the ability to control nodulation, besides having antifungal activity and acting as precursors of phytoalexins [19]. The main sources of isoflavones are legumes from the family *Fabaceae* (e.g., soybean and red clover) [20].

### 2.4. Anthocyanidins

Anthocyanidins are aglycones species of their respective glycosides called anthocyanins. Anthocyanins chemically occur as glycosides of flavylium (2-phenylbenzopyrylium) salts differing from them by structural variations in the number of hydroxyl groups, degree of methylation of the hydroxyl groups, and the number of sugar moieties bonded to the structure [21]. They are distributed in plums, cherries, and berries of several plants, as pigments responsible for the color [21].

### 2.5. Flavanones

Flavanones’ general structure is characterized by the presence of a carbonyl group in the C4 position, the absence of substituents in the C3 position, and a double bond between C2 and C3 [22]. They usually occur as glycosides, usually rutinosides (6-*O*-α-L-rhamnosyl-D-glucosides) and neohesperidosides (2-*O*-α-L-rhamnosyl-D-glucosides) [23]. Flavanones are widely distributed in plants, especially in *Compositae*, *Leguminose*, and *Rutaceae* [22]. Citrus fruits are the major dietary source of flavanones: indeed, lemon, lime, mandarin and sweet orange are rich in rutinosides, while grapefruit and sour orange are rich in neohesperidoses [23].

### 2.6. Flavonols

Flavonols’ chemical structure is characterized by the presence of a carbonyl group in the C4 position, a C2-C3 double bond, and a hydroxy group bonded to the carbon in the C3 position [24]. Flavonols are mainly found in fruits and vegetables (e.g., apples and onions), as well as in beverages (e.g., red wine and teas) [25]. They are usually found in plants bound to sugars as *O*-glycosides [26]. They are responsible for color, taste, and the prevention of substances such as vitamins and enzymes from oxidation, as well as for protection against ultraviolet radiations and parasites [24].

### 2.7. Chalcones

Chalcones have a common chemical structure, 1,3-diaryl-2-propen-1-one, also known as “chalconoid” [27]. The structure exists as *trans* and *cis* isomers, with the second isomer being more thermodynamically stable than the first one. They can be found in vegetables (e.g., tomatoes), fruits (e.g., sweet oranges, apples, licorice, and Chinese ginger), and beverages (e.g., red wine, beer, coffee, and teas) [28].

### 2.8. Bioactivity and Bioavailability of Flavonoids

Flavonoids present many different biological activities, as will be discussed in detail in the next sections, including antioxidant, antiaging, anti-inflammatory, immunomodulatory, cardioprotective, antimicrobial, antiviral, antibacterial, antiparasitic, antifungal, and anticancer activities [29]. According to the U.S. Food and Drug Administration (FDA), the definition of bioavailability is “the rate and extent to which the active ingredient or active moiety is absorbed from a drug product and becomes available at the site of drug action” [30]. The bioavailability of flavonoids is generally low. In addition, it usually depends on flavonoid classes, but it can also vary among individual compounds within a particular class [31]. Flavonoid bioavailability depends on many properties, such as their molecular weight, chemical structure, and degree of glycosylation, as well as the type of sugar moieties [31,32]. The solubility of flavonoids in water is generally very low; as a consequence, their absorption by oral administration is difficult, thus leading to low bioavailability and low therapeutic effect [33,34,35]. Furthermore, flavonoids have low stability, are easily degraded in acidic medium, and have a high metabolic rate [35,36]. Many strategies have been explored to increase flavonoid bioavailability and metabolic stability, such as the formation of microemulsions or flavonoid methylation [31], as well as their encapsulation in a drug delivery system, as one of the most promising approaches.

## 3. Polymeric Systems for Controlled Drug Release

A polymeric drug delivery system can be defined as a formulation or a device that enables the introduction of a therapeutic substance into the body, improving its safety and efficacy by controlling the rate, time, and place of its release in the body [37]. Controlled drug delivery strategies significantly contribute to transforming promising substances into successful therapies [38].

**Figure 2:**
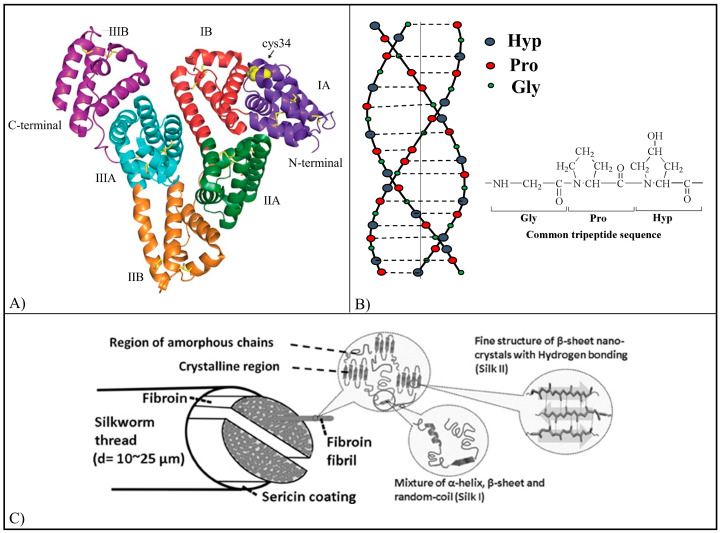
Chemical structure of proteins for drug release. (**A**) Crystal structure of human serum albumin (HSA); the illustration shows the tertiary structure of HSA complexed with stearic acid (PDB 1e7e), the six domains that characterize albumin structure are shown in purple (IA), red (IB), green (IIA), orange (IIB), blue (IIIA), and violet (IIIB), yellow sticks depicture disulfide bridges, and yellow spheres highlight the available cysteine 34 in domain IA (reproduced with permission from [42], BioMed Central, 2016). (**B**) Triple chain structure of collagen (Col) fibrils and chemical structure of the most common tripeptide sequence found in Col, composed of glycine (Gly), proline (Pro) and hydroxyproline (Hyp) sequences (reproduced with permission from [43], Elsevier, 2010). (**C**) Schematic of silk fibroin structure (insets showing the overall fibril structure and the fine β-sheet antiparallel alignment of polypeptide chains). Reproduced with permission from [44], Wiley, 2015.

Different polymers of natural and synthetic origin have been employed for the development of drug delivery systems. Polymers from natural sources include proteins, polysaccharides, and microbial polyesters [39]. Proteins are natural polyamides constituted by secondary structures (α-helix and β-foils), and most of them are characterized by the ability to self-assemble into complex 3D hierarchical architectures. Proteins show particular mechanical, chemical, electrical, electromagnetic, and optical properties [39]. Thanks to their properties, various proteins such as human serum albumin (HSA) (Figure 2A), collagen (Col) (Figure 2B), gelatin (Gel), silk fibroin (SF) (Figure 2C), and zein have been studied for application in drug delivery [40,41].

**Figure 3:**
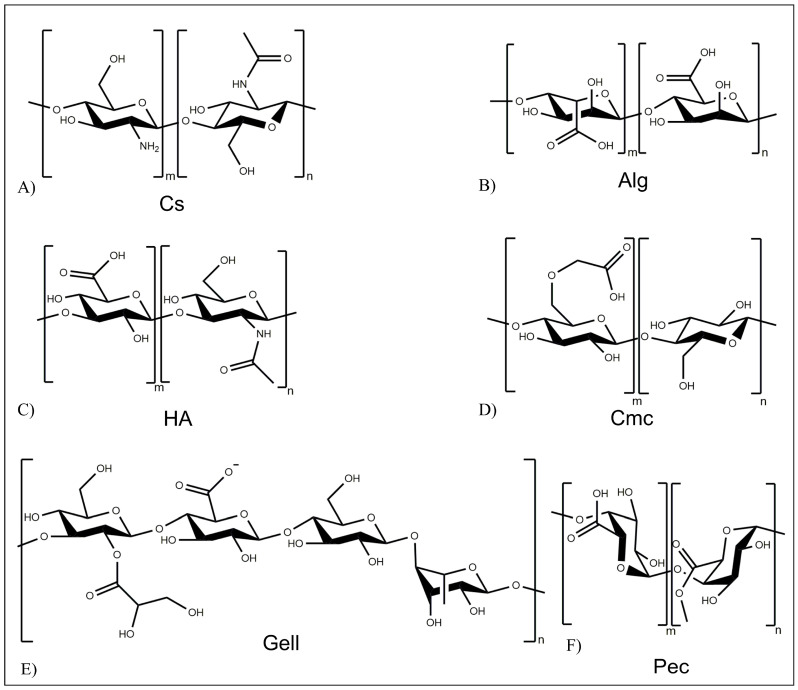
Chemical structure of polysaccharides for drug release: (**A**) chitosan (Cs), (**B**) alginic acid (Alg), (**C**) hyaluronic acid (HA), (**D**) carboxymethylcellulose (CMC), (**E**) gellan gum (Gell), (**F**) and pectin (Pec).

Polysaccharides are based on mono- or disaccharide repeating units linked by glycosidic bonds [45]. Their peculiar physicochemical and biological properties, such as biocompatibility, biodegradability, and low immunogenicity, make them suitable for drug delivery applications. Numerous polysaccharides also have the capability to adhere to the mucus layer covering epithelial surfaces throughout the human body. Thanks to this property, polysaccharide-based carriers can extend drug residence time in the gastrointestinal tract, increasing its bioavailability. Many polysaccharides, e.g., chitosan (Cs) (Figure 3A), alginate (Alg) (Figure 3B), hyaluronic acid (HA) (Figure 3C), cellulose, carboxymethylcellulose (CMC) (Figure 3D), gellan gum (Gell) (Figure 3E), and pectin (Pec) (Figure 3F), have been employed for the development of drug delivery systems [45].

**Figure 4:**
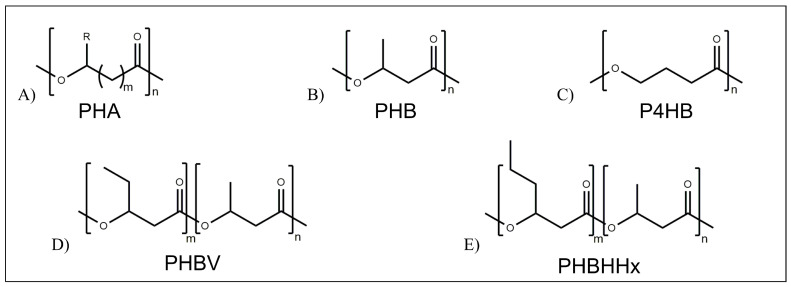
Chemical structure of PHA for drug release: (**A**) general structure; (**B**) poly(3-hydroxybutyrate) (PHB); (**C**) poly(4-hydroxybutyrate) (P4HB); (**D**) poly(3-hydroxybutyrate-*co*-3-hydroxyvalerate) (PHBV); (**E**) poly(3-hydroxybutyrate-*co*-3-hydroxyhexanoate).

Microbial polyesters are represented by polyhydroxyalkanoates (PHA) which are naturally produced by certain types of bacteria as an energy source during periods of unstable growth [46,47]. PHA are aliphatic polyesters with a variable number of carbon atoms in the monomeric units (Figure 4A) [47]. A wide range of PHAs with different numbers of C atoms in the repeating unit, e.g., poly(3-hydroxybutyrate) (PHB) (Figure 4B), poly(4-hydroxybutyrate (P4HB) (Figure 4C), poly(3-hydroxybutyrate-*co*-3-hydroxyvalerate) (PHBV) (Figure 4D), and poly(3-hydroxybuthyrate-*co*-3-hydroxyexanoate) (PHBHHx) (Figure 4E), allow tuning the physical-chemical and mechanical properties of the resulting biomaterial. PHAs are fully biocompatible and non-immunogenic and do not generally cause any inflammation reactions [48]. However, FDA has not yet approved any PHA-based nanomedicines for clinical applications, and their use is still limited to the experimental stage [46,48].

**Figure 5:**
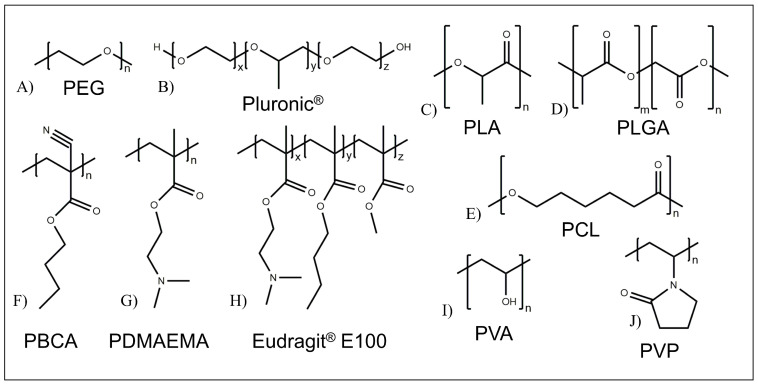
Chemical structure of representative synthetic polymers: (**A**) poly(ethylene glycol) (PEG), (**B**) poloxamer (Pluronic^®^), (**C**) poly(lactic acid) (PLA), (**D**) poly(lactic-*co*-glycolic acid) (PLGA), (**E**) poly(ε-caprolactone) (PCL), (**F**) poly(butyl cyanoacrylate) (PBCA), (**G**) poly(2-dimetylaminoethyl methacrylate) (PDMAEMA), (**H**) Eudragit^®^, (**I**) poly(vinyl alcohol) (PVA), (**J**) poly(vinyl pyrrolidone) (PVP).

Polyethers, and in particular poly(ethylene glycol) (PEG) (Figure 5A), represent one of the most important classes of synthetic polymers employed for the development of drug delivery systems. PEG, originally described in 1859, displays unique osmotic properties and high solubilities in both aqueous and organic solvents [38]. It is relatively safe for use in humans and widely used as a protein-conjugation polymer. PEGylation technique, i.e., functionalization of other polymers with PEG, has become very important since it permits the extension of the circulation half-life of many proteins and reduces their immunogenicity [38]. Poloxamers (Figure 5B), available also under the trademark Pluronics^®^ (BASF), are water-soluble nonionic A-B-A and B-A-B triblock copolymers, where A is poly(ethylene oxide) (PEO), and B is poly(propylene oxide) (PPO) [49]. The monomeric units of the two copolymer blocks are chemically dissimilar (e.g., polar and non-polar), making these block copolymers amphiphilic and leading to surface active properties. They are generally employed for the production of micelles with a hydrophobic core favoring the encapsulation of hydrophobic drugs and a hydrophilic shell favoring the interaction of the systems with water and their stability in the physiological environment [49].

Synthetic aliphatic polyesters mainly comprise poly(α-hydroxy acids) (Figure 5C,D) and poly(ε-caprolactone) (PCL) (Figure 5E). Poly(lactic acid) (PLA) (Figure 5C), poly(glycolic acid) (PGA), and their copolymers poly(lactic-*co*-glycolic acid) (PLGA) (Figure 5D), are poly(α-hydroxy acids) approved for clinical applications thanks to their biocompatibility, controlled biodegradability, processing versatility and suitable mechanical properties [39]. PCL is a semicrystalline polyester with thermoplastic behavior. It has been widely investigated for biomedical applications since its biocompatibility, slow in vitro degradation, viscoelastic properties that are suitable for many different fabrication techniques, and good blend compatibility [39].

Vinyl-based polymers are also employed in an increasing number of higher-added-value and specialty applications, including in the area of biomaterials (for example, drug delivery devices and tissue engineering scaffolds) [50]. Advanced polymerization processes allow a high degree of tunability of polymer properties and functionalization. As a consequence, the past few years have been marked by an increased interest in the design of innovative and more sophisticated vinyl molecules [50]. Some vinyl (co)polymers, e.g., poly(butyl cyanoacrylate) (PBCA) (Figure 5F), poly(2-(dimethylamino)ethyl methacrylate) (PDMAEMA) (Figure 5G), Eudragit^®^ (Figure 5H), poly(vinyl alcohol) (PVA) (Figure 5I), poly(vinylpyrrolidone) (PVP) (Figure 5J), poly(acrylic acid) (PAA), poly(methacrylic acid) (PMAA), present mucoadhesive properties, allowing the adhesion of the drug delivery system to mucosal surfaces (e.g., buccal and gastrointestinal), thus guaranteeing a prolonged permanence of the drug in blood circulation and high absorption capacity across the small intestine [51]. Moreover, many vinyl monomers have been reported as pH-responsive materials, namely, ethyl methacrylate (EMA), MMA, MAA and DMAEMA. The resultant cationic or anionic (co)polymers can show a stimuli-responsive drug release exploitable on the basis of pH differences between the oral cavity (pH 5.8 to 7.4), the stomach (pH 1.0 to 3.5) and the intestine (pH 7.0) [51].

**Figure 6:**
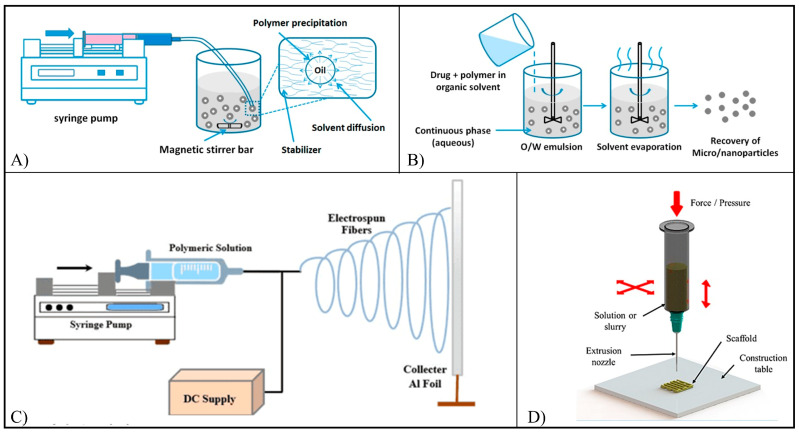
Schematic representation of (**A**) nanoprecipitation method (reproduced from [54], MDPI, 2016); (**B**) emulsification method (reproduced from [54], MDPI, 2016); (**C**) electrospinning (reproduced with permission from [58], Lifescience Global, 2016), and (**D**) additive manufacturing. Reproduced with permission from [59], Wiley, 2015.

Different typologies of drug delivery systems have been employed for flavonoid loading (Figure 6), e.g., nanoparticles (NPs), also known as nanospheres and constituted by a solid matrix system in which the drug is dispersed, nanocapsules (NCs), constituted by a polymeric shell surrounding a drug-containing cavity (core), nanomicelles (NMs), which are obtained by amphiphilic block copolymers that self-assemble forming nanospheres, and scaffolds for tissue engineering, which are 3D porous structures designed to favor the regeneration of biological tissues upon implantation in the patient [52,53]. The most employed systems for the delivery of flavonoids are NPs, mainly obtained by means of a nanoprecipitation (Figure 6A) or emulsification method (Figure 6B). Synthesis of NPs through nanoprecipitation involves the dissolution of the polymer and the drug in a water-miscible organic solvent and the slow addition of the organic phase into an aqueous solution containing colloidal stabilizers under moderate stirring [54]. The formation of the NPs is instantaneous and due to the diffusion of the organic solvent into the aqueous phase causing the precipitation of the polymer and the nucleation of the particles [54]. The emulsion method consists of the formation of binary (oil/water) or ternary emulsions (water/oil/water) between the organic phase containing the polymer and the aqueous phase containing the stabilizer, followed by the evaporation of the organic solvent [55]. In the first case, a hydrophobic drug is dissolved in the organic phase together with the polymer, while in the second case, the hydrophilic drug is dissolved in water, and the resulting solution is emulsified with the organic phase. In both cases, the last step involves emulsifying in an aqueous phase [55].

Flavonoid-loaded scaffolds are typically fabricated by electrospinning (Figure 6C) or additive manufacturing (Figure 6D). Electrospinning involves the extrusion of a polymer/drug solution through a needle and the electrification, by means of a high voltage power supply, of the pendant droplet, which is deformed into a cone (Taylor cone) [56]. A charged polymer solution jet ejected from the tip of the cone undergoes whipping motions, stretching, and solidification due to solvent evaporation, resulting in solid nano/microfibers, which are collected on a conductive collector to form non-woven meshes (MHs) [56]. Additive manufacturing techniques are based on computer-aided design and manufacturing of 3D objects layer-by-layer through different approaches (e.g., melt- or solution-extrusion). They allow the fabrication of scaffolds with customized shapes and dimensions, as well as the architecture of their porous structure [57].

## 4. Flavonoid-Based Commercial Products 

Despite all the beneficial effects attributed to flavonoids, there are still many challenges to their approval and employment as drugs for clinical application. The main issues concern their isolation and purification, as well as epidemiological and pharmacokinetics aspects [60]. Since flavonoids are present at very low levels in natural sources, their extraction and isolation to meet the needs of drug mass production are challenging aspects [61]. Moreover, the extraction techniques typically employed (e.g., solvent extraction, column chromatography, and high-performance liquid chromatography) are based on high-cost and time-consuming methods that usually result in low yields [60]. Epidemiological challenges are related to the cost and time necessary to determine the potential therapeutic effect on individuals that have assumed dietary flavonoids. Available data on this aspect are often controversial and inconsistent [62,63]. Pharmacokinetics issues are usually associated with low flavonoid bioavailability and oral absorption, as explained above.

Although all the aforementioned shortcomings, flavonoids are already employed in the formulation of many commercial drugs [64]. The most used are glycosylated flavonoids, such as rutin, diosmin, and hesperidin. Rutin is found in drugs used for the relief of pain and inflammation caused by degenerative conditions, rheumatoid arthritis, surgery, or everyday life (e.g., Rosehip^®^, Rutosin^®^, Wobezym^®^, Phlogenzym^®^, Rutozym^®^) or in food supplements (Vitamin C Plus^®^). Diosmin is found in drugs used for hemorrhoids (e.g., Analpram^®^) or in combination with hesperidin in drugs used for chronic venous insufficiency, varicose veins, hemorrhoids, and other conditions affecting the vascular tissue (e.g., Daflon^®^_,_ Dioflex^®^, Ruventin^®^, Aveflon^®^, Davmorid^®^, Dioscomb^®^).

## 5. Flavonoid-Loaded Polymeric Systems

An updated overview of the literature on polymeric systems for the controlled release of flavonoids is reported in this section. In particular, the presentation and discussion of published articles on this topic are reported for each investigated flavonoid. The most investigated flavonoids for drug delivery systems (DDS) are reported in Table 1, highlighting their application and the most important outcomes. 

**Figure 7:**
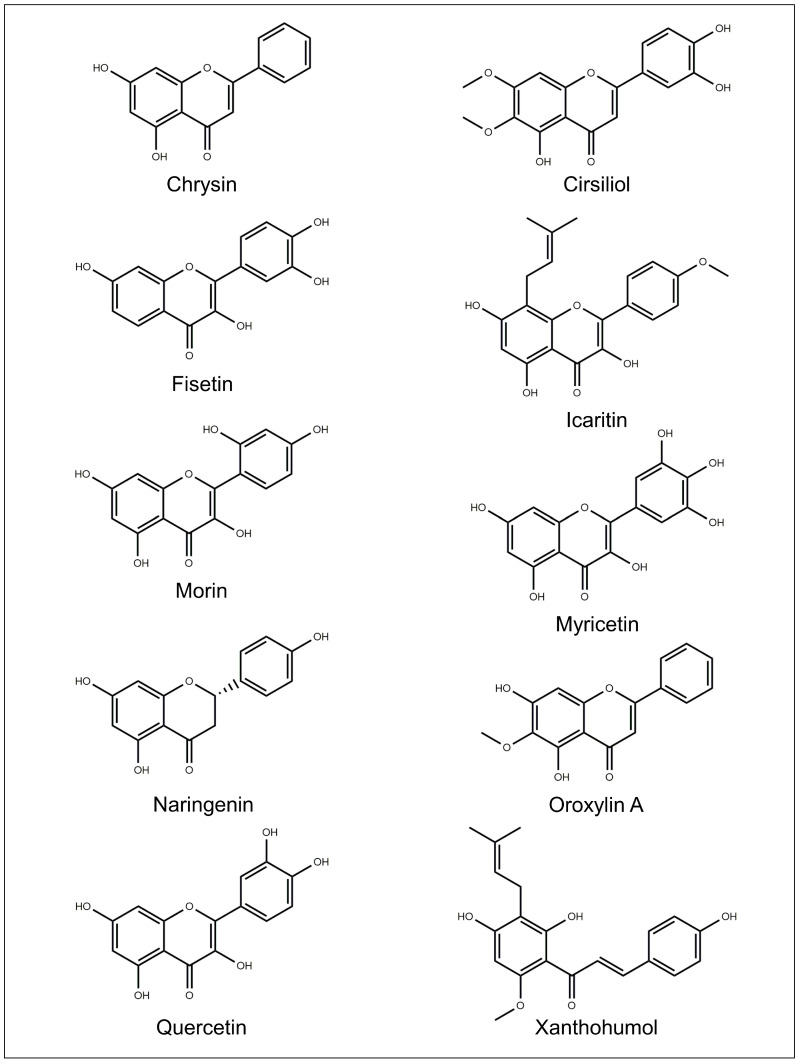
Chemical structure of the aglycone flavonoids encapsulated into a polymeric matrix.

**Figure 8:**
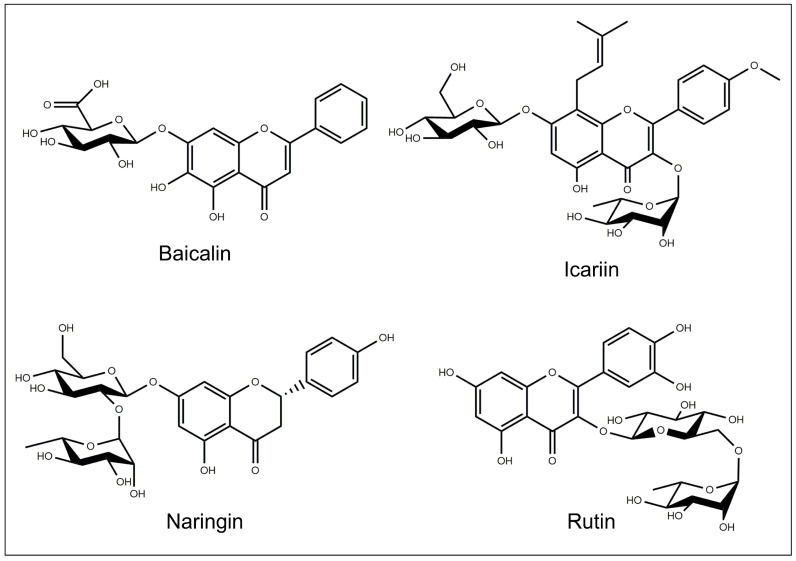
Chemical structure of the glycosylated flavonoids encapsulated into a polymeric matrix.

The chemical structures of the flavonoids investigated for drug delivery systems are depicted in Figure 7 and Figure 8.

### 5.1. Baicalin

Baicalin (Bai) (5,6-dihydroxy-4-oxo-2-phenyl-4H-chromen-7-yl beta-D-glucopyranosiduronic acid) (Figure 8) is one of the major bioactive flavone glucuronides present in the radix of *Scutellaria baicalensis* [65,66]. It is used in traditional Chinese medicine as a remedy for the treatment of inflammations [67], fever, allergic diseases and cancer [65].

**Figure 9:**
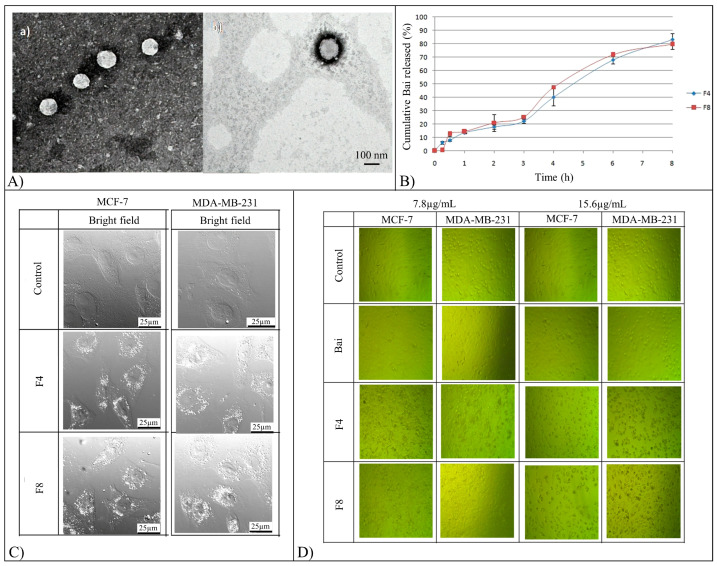
(**A**) Transmission Electron Microscopy (TEM) micrographs of the selected formulations F4 (**a**) and F8 (**b**); (**B**) Cumulative % of released baicalin (Bai) over a period of 8 h; (**C**) Bright-field images of cells incubated for 4 h with the two formulations, and (**D**) Morphological appearance of cells treated with two concentrations of free and encapsulated Bai. Reproduced with permission from [68], Springer Nature, 2019.

Bai-loaded PLGA polymeric nanocapsules (NCs) were formulated using the nanoprecipitation technique and two different tensioactives (F4 Tween^®^ 80 and F8 Poloxamer P407) [68]. The NCs were characterized by a 169 to 215 nm oily core constituted by the corn oil pegylated ester (Figure 9A). Bai encapsulation efficiency of the NCs ranged from 85 to 94% thanks to Bai’s high solubility in the oily phase. The in vitro release of Bai followed zero-order kinetics (Figure 9B) for the two NCs formulations tested. The anti-breast cancer activity of the prepared NCs was determined by employing MCF-7 and MDA-MB-231 cells. Both F4 and F8 formulations achieved adequate cellular localization after 4 h of incubation (Figure 9C). Differently from free Bai treatment, loss of integrity, condensation of cellular materials and decrease of adherent cells in a dose-dependent manner were observed in the case of F4 and F8 formulations. F4 formulations showed higher apoptotic effects on cancer cells than F8, probably due to the superior cellular accumulation caused by the smaller particle size and by the different surfactant composition (Figure 9D).

Nanohydrogels made of gellan functionalized with cholesterol, a hydrophobic molecule, were employed as a self-assembly system in water, capable of encapsulating hydrophobic molecules, such as Bai. The gellan-cholesterol system was dispersed in phosphate buffer solution (PBS) in the presence of Bai and sonicated or autoclaved to produce particles with a mean diameter of 350 nm or larger than 500 nm, respectively [69]. Both kinds of nanohydrogel results were compatible in vitro with 3T3 fibroblasts. The nanohydrogels were also applied in the back of rats treated with 12-O-tetradecanoylphorbol 13-acetate (TPA), and they resulted in healing TPA-provoked lesions and inhibiting markers of inflammation more efficiently than free drugs or dexamethasone (an anti-inflammatory drug) [69].

Bai-loaded nanomicelles (NMs) made of Pluronic F68-SA conjugate were investigated for human lung cancer treatment [70]. NMs with an average diameter of 106.2 nm and Bai loading of 84.3% were prepared by solvent evaporation and rehydration method. They reduced the in vitro viability of A549 cells in a time- and dose-dependent manner determining both cell apoptosis and necrosis, with increased cytotoxic effects in comparison with free Bai.

Different types of vesicles prepared by the thin film hydration method, including liposomes, penetration enhancer vesicles, and transfersomes, were evaluated to deliver Bai in the ocular systems [71]. The obtained vesicles presented average diameters ranging from 667 to 1341 nm and encapsulation efficiency values ranging from 26 to 99%. The cumulative percentage release of Bai from the different vesicle systems over a period of 8 h was in the range of 46 to 89%. All Bai vesicular systems resulted in being non-irritant and presented good ocular tolerance in a rabbit model. Pharmacokinetics parameters indicated that the transfersomes results showed they were the fastest to be absorbed, while liposomal formulations presented the highest Bai maximum concentration and total amount released [71].

Transfersomes were also used as carriers for skin delivery of Bai after combination with gellan-cholesterol derivative to improve their viscosity and skin delivery capabilities [72]. Bai-loaded transfersomes presented an average diameter of 80 nm and encapsulation efficiency of 37%, while after the formation of the gellan transfersomes complex, the two values increased to 123 nm and 45%, respectively. Release studies carried out using a newborn pig skin model demonstrated that encapsulating Bai in transfersomes and gellan transfersomes significantly increased its accumulation in the stratum corneum and dermis. Lesions healing and inhibition of the inflammation markers (TNFα and IL-1β) were also enhanced in comparison to what was achieved with a commercial betamethasone steroid cream [72].

### 5.2. Cirsiliol

Cirsiliol (Cir): (2-(3,4-dihydroxyphenyl)-5-hydroxy-6,7-dimethoxy-4H-chromen-4-one) (Figure 7) is a flavonoid first isolated from *Achillea fragrantissima* [73]. It presents a great variety of biological functions, such as antibacterial, antiproliferative, and anti-inflammatory activity [73].

Cir oil-cored PEG-PCL NCs with a mean diameter of 158.1 nm and a loading of 53.5% were prepared by employing the nanoprecipitation method [74]. In vitro, Cir release followed a typical biphasic profile, with a fast release phase within the first 24 h and a sustained release thereafter. The slow release kinetics observed was attributed to the diffusion of Cir through the shell containing PCL, characterized by high crystallinity degree and hydrophobicity. Cir-loaded NCs had an antiproliferative effect against MCF-7 breast cancer cells even if free Cir was significantly more toxic [74].

### 5.3. Chrysin

Chrysin (Chr): (5,7-Dihydroxy-2-phenyl-4H-chromen-4-one) (Figure 7) is a natural flavonoid abundantly present in many plant extracts, including propolis, blue passion flower (*Passiflora caerulea*), and honey [75]. It shows antidepressant [76], antioxidant [77], anti-inflammatory [78], antidiabetic [79], antiallergic [80], antibacterial [81], hepatoprotective [77], anticancer [82], and neuroprotective activities [83].

PLGA NCs loaded with Chr were investigated for potential application in the management of diabetes and hyperlipidemia [84]. The capsules with a mean diameter ranging between 145 and 284 nm, encapsulation efficiency between 75.6 and 89.7%, and drug loading between 2.5 and 17.4% were obtained employing a nanoprecipitation method. Chr release profiles showed an initial burst release phase followed by a sustained release phase. After 24 h, the amount of Chr release ranged from 17.2% to 52.8% depending on the surfactant concentration and on the particle size, affecting the surface area to volume ratio for drug release. The prepared NCs were studied as antihyperlipidemic agents on high-fat diet-treated adult male albino rats. After 28 d of treatment with Chr-PLGA NCs, rats showed increased levels of high-density lipoprotein (from 15.3 to 54.0 mg/dL) and reduced levels of low-density lipoprotein and triglycerides (88 and 71% of reduction, respectively) differently from rats treated with Chr suspension. Chr NCs were also tested for their antidiabetic properties on hyperglycemic rats. Serum blood glucose level was reduced by 46% 24 h after treatment with Chr NCs, against a 30% reduction in the first 3 h and no appreciable decrease after 24 h in animals treated with Chr suspension. This superior performance was probably due to the encapsulation of Chr, which increased its bioavailability, target efficacy, protection from in vivo degradation, permeability across the intestinal membrane, and residence time in the intestinal cavity [84].

Chr-loaded PLGA-poly(ethylene glycol) (PEG) copolymer NPs with an average diameter in the range from 70 to 300 nm were prepared by a double emulsion and solvent evaporation method, achieving an encapsulation efficiency of 98.6% and a Chr loading of 16.2% [85]. Encapsulation improved Chr cytotoxic effect against human gastric carcinoma cells (HGCCs), determining cell growth inhibition and the decline of the expression of microRNA (miR) [85]. Besides suppressing miR harmful to patient health, Chr can increase the expression of miR, such as miR-9 and Let-7, which are tumor suppressors [86]. This effect was studied employing Chr-loaded PLGA-PEG NPs prepared by a single emulsion solvent evaporation technique, showing an average diameter in the range of 40 to 70 nm, 98.6% encapsulation efficiency and 16.2% loading. NPs were able to upregulate the expression of miR-9 and Let-7 in HGCCs at a higher level than free Chr [86]. PLGA-PEG copolymer was also employed for the preparation of Chr-loaded core-shell NPs with a mean diameter in the range of 70 to 300 nm through a double emulsion technique [87]. They showed an increased anti-growth effect on the breast cancer cell lines T47D and MCF-7 in comparison to free Chr [87].

Combinatorial therapy is an important strategy to overcome multidrug resistance in cancer cells by targeting different signaling pathways. Chr and curcumin (Cur) were co-encapsulated in PEGylated PLGA NPs with a diameter from 210 to 258 nm, achieving encapsulation efficiencies of Cur and Chr of 78.3 and 83.5% and loading capacity of 12.6 and 10.3%, respectively [88]. In vitro release kinetics investigation demonstrated that the release of Chr was faster in the first 12 h and then slower than that of Cur. The in vitro cytotoxicity and the synergistic anticancer effect of the two flavonoids were evaluated employing Caco-2 cells. In particular, Cur exerted higher cytotoxicity when encapsulated, while Chr exerted higher cytotoxicity in the free form. In addition, combined Cur and Chr formulations exerted higher cytotoxicity when encapsulated [88].

PLGA-PEG-PLGA triblock copolymer NPs dual-loaded with 5-fluorouracil (5-Fu) and Chr were obtained employing a double emulsion evaporation method [89]. NPs had a mean diameter of 40 nm, the encapsulation efficiency of 5-Fu and Chr was 81.3 and 97.5%, respectively, while their loading was 8.1 and 9.8%. In vitro drug release kinetics investigation in physiological conditions demonstrated that the developed NPs were characterized by an initial burst release phase (20 and 12% of 5-Fu and Chr, respectively, released within the first 5 h) followed by a sustained release phase. After 96 h of incubation, respectively, 59% of 5-Fu and 62% of Chr were released. Reducing the pH of the release medium at 5.4 increased the release of 5-Fu (80% released after 96 h) but did not affect Chr release kinetics. This could be related to the lack of ionic interactions between Chr and the copolymer. Increasing the temperature to 40 °C resulted in an increase in the release rate of both drugs caused by the thermoresponsive properties of the copolymer. The anticancer activity of the dual-loaded NPs was determined by employing the HT29 cell line as a model of colon cancer cells. The IC_50_ of dual-loaded NPs resulted in being significantly lower in comparison with free drugs and unloaded NPs. The induction of cancer cell apoptosis was confirmed by DAPI staining, performed after 48 h of treatment, which showed chromatin fragmentation in cells treated with NPs [89].

A PLGA-PEG tri-block copolymer was employed for the preparation of Chr-loaded NPs using the oil-in-water emulsion solvent evaporation technique [90]. NPs with a mean diameter of 205 nm, encapsulation efficiency of 88% and drug loading of 3.7% were obtained. In vitro release kinetics was studied at different pH values. Chr release profile at pH 7.4 was characterized by a burst release phase within the first 8 h followed by a sustained release phase during the next 7 days. About 65% of loaded Chr was released from the NPs during the first 120 h. At pH 4.4, NPs showed a higher release rate, with more than 50% of the loaded drug being released in the first 48 h. The obtained Chr-loaded NPs showed the ability to re-polarize peritoneal macrophages from a pro-inflammatory phenotype to an anti-inflammatory one. This effect can support tissue repair and reconstruction, cell replacement, and matrix remodeling [90].

**Figure 10:**
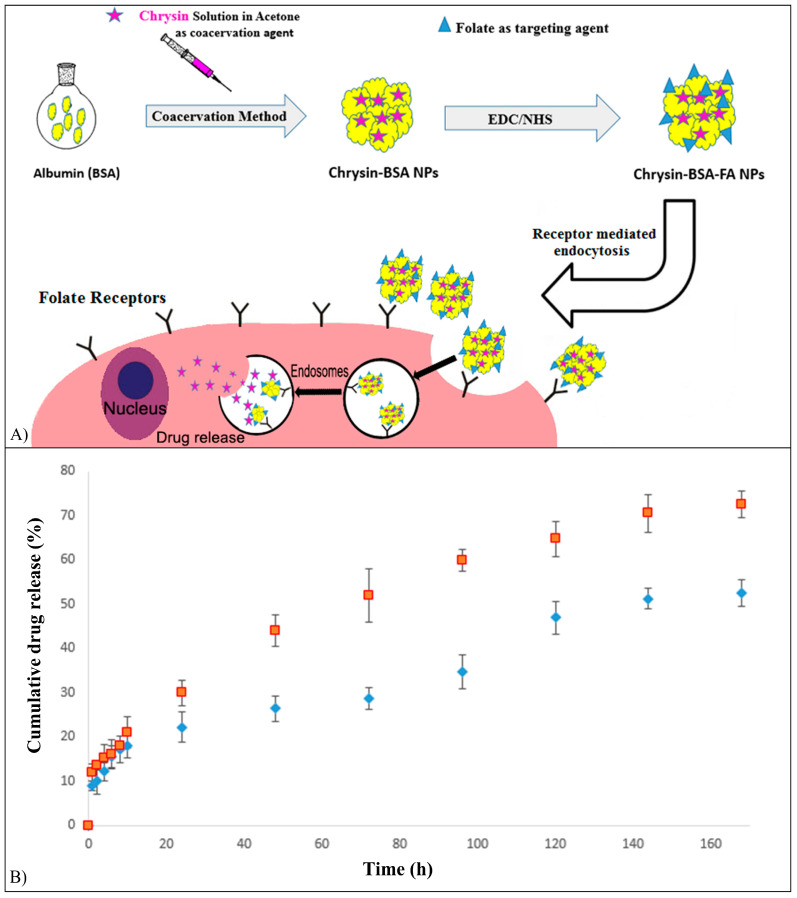
(**A**) Schematic representation of the synthesis of Chr-loaded FA-BSA NPs and receptor-mediated endocytosis; (**B**) Cumulative Chr release profiles, blue symbol pH 7.4, red symbol pH 5.8. Reproduced with permission from [91], Elsevier, 2018.

Chr-loading in bovine serum albumin (BSA) NPs functionalized with folic acid (FA), which is a natural ligand of folate receptors that are upregulated in malignant cells, was investigated to achieve efficient solubility, bioavailability, and tumor targeting [91]. The Chr-loaded NPs were prepared using a desolvation method, and the functionalization of the NPs surface was carried out by dropping a dimethyl sulfoxide solution of FA, 1-Ethyl-3-(3-dimethylaminopropyl) carbodiimide and N-hydroxy succinimide in the dispersion of the particles (Figure 10A). The average NPs size was 90.6 nm, and Chr loading was 2.1%. In vitro release kinetics was studied for 168 h in PBS at pH values of 7.4 and 5.8 (Figure 10B). Chr-loaded NPs showed an initial sustained and controlled release (20% of Chr released in 10 h at pH 7.4 and 5.8), an ensuing release (35% and 57% of Chr released after 96 h at pH 7.4 and 5.8, respectively), and a plateau level after 120 h. Chr release rate from FA-BSA NPs increased in an acidic medium, typical of the tumor microenvironment. FA functionalization increased the in vitro cytotoxic effect of Chr-loaded NPs against MCF-7 cells [91].

**Figure 11:**
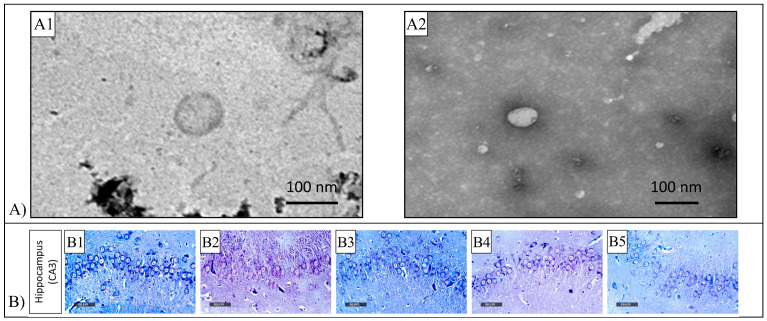
(**A**) Transmission electron microscopy (TEM) micrographs of (**A1**) transfersomal formulation T1 and (**A2**) chitosan (Cs) composite vesicle; (**B**) Effect of chrysin formulae on doxorubicin (Dox)-mediated neurodegenerative changes using toluidine blue staining. Representative photomicrographs for the control group (**B1**), Dox-treated group (2 mg/kg) (**B2**); Chr transfersomal-treated group (0.5 mg/kg) (**B3**); Chr chitosan composite vesicle-treated group (0.5 mg/kg) (**B4**); Chr oral administration (30 mg/kg) (**B5**); of stained sections of hippocampal CA3 regions (scale bar 50 μm). Reproduced with permission from [92], Elsevier, 2021.

The antioxidant activity of Chr can be exploited in reducing the oxidative stress and inflammation induced by other drugs, such as Doxorubicin (Dox) which is one of the most used chemotherapeutic agents [92]. For this purpose, Chr-loaded transfersomes prepared by an ethanol injection method (Figure 11A) with 156.4 nm particle size and 95.8% encapsulation efficiency were investigated together with chitosan (Cs) complexed vesicles which presented an average particle diameter of 617.2 nm and encapsulation efficiency of 97.4%. In vitro release study revealed that over 120 h, both formulations presented a sustained drug release with a maximum Chr cumulative release of 58.0% and 46.8% for transfersomes and vesicles, respectively. In vivo experiments carried out on male Sprague-Dawley rats showed that the administration of Chr-loaded vesicles via intranasal route led to the same or superior effect on memory acquisition and spatial memory, in comparison with orally-administrated Chr. Moreover, loaded vesicles corrected neurodegeneration induced by Dox (Figure 11B) [92].

PCL-PEG-PCL block copolymer nanofibrous scaffolds were fabricated by electrospinning and loaded with Chr since its antioxidant activity can also be employed to increase the proliferation and the preservation of human adipose-derived stem cells (ADSCs) for regenerative medicine applications [93]. The block copolymer was synthesized by ring opening polymerization of ε-CL using PEG as initiator and stannous octanoate as catalyzer. A polymer concentration in the feeding solution of 10 %*w/v* gave rise to bead-free nanofibrous meshes (MHs) with an average diameter ranging from 300 to 400 nm. ADSCs cultured on the Chr-loaded mats showed higher viability and expression levels of stemness genes in comparison to what was observed with non-loaded MHs and the control.

Chr was encapsulated into freeze-dried PCL-Gel scaffolds for endodontic tissue engineering to exploit its antimicrobial, anti-inflammatory and mineralization activity [94]. The Chr-loaded scaffolds showed increased antimicrobial activity against *Acinetobacter baumannii*, *Pseudomonas aeruginosa*, *Staphylococcus aureus*, and *Enterococcus faecalis* strains, as well as an increased dental pulp stem cells (DPSCs) viability in comparison to non-loaded PCL-Gel scaffolds. In addition, Chr-loaded scaffolds led to a significant reduction of the TNFα and DCF levels of the DPSCs, demonstrating their anti-inflammatory and antioxidant activity, as well as higher alkaline lysis phosphatase (ALP) and Alizarin Red S (ARS) activity related to cell differentiation and mineralization.

Chr also presented osteogenic properties which can be optimized through its controlled release [95]. Chr-loaded Cmc/Cs/HA scaffolds were prepared by ionic gelation employing a 1-ethyl-3-(3-dimethylaminopropyl)carbodiimide hydrochloride solution, followed by freeze-drying. The scaffolds were characterized by the presence of a spread and interconnected porosity with a pores dimension of 156 µm. Chr addition did not alter scaffolds’ pore size, as well as their swelling, degradation, and protein absorption behavior. In vitro, the Chr release profile presented an initial sustained release phase followed by a steady state release up to 13 days after the test. In vitro cell culture test demonstrated that Chr loading resulted in increased proliferation and differentiation towards osteoblasts of mesenchymal stem cells (MSCs) cultured on the scaffolds [95].

### 5.4. Fisetin

Fisetin (Fis): (2-(3,4-Dihydroxyphenyl)-3,7-dihydroxy-4H-chromene-4-one) (Figure 7) is a flavonoid found in vegetables (onions and cucumbers), fruits (persimmon, apples, strawberries), wine, and nuts [96]. It is characterized by anticancer [96], anti-inflammatory [97], antibacterial [98], antidepressant [99], and antioxidant [100] activity.

**Figure 12:**
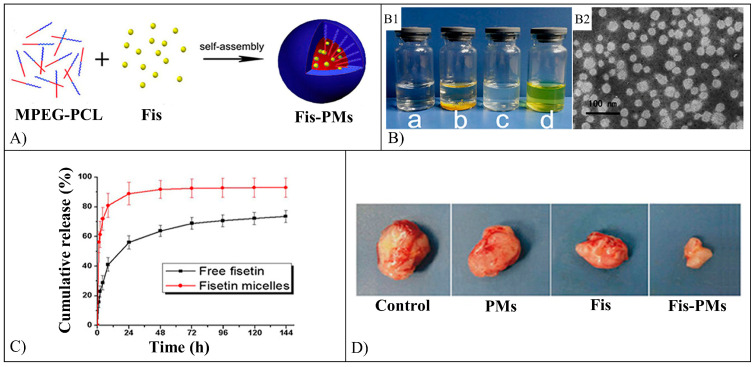
(**A**) Schematic representation of the preparation of Fis-loaded MPEG-PCL PMs; (**B**) (**B1**) representative pictures of (a) water, (b) Fis in water, (c) blank PMs in water, (d) Fis-loaded PMs in water, (**B2**) TEM images of Fis-loaded PMs; (**C**) in vitro release data for free and encapsulated Fis, and (**D**) representative photographs of subcutaneous tumors subjected to different treatments. Reproduced with permission from [101], ACS Publications, 2015.

Fis-loaded PEG-PCL NMs were prepared to employ a self-assembly method (Figure 12A) and were investigated for their anticancer activity [101]. The mean diameter of the obtained NMs was 22.4 nm (Figure 12B), with an encapsulation efficiency of 98.5% and a drug loading of 9.9%. The in vitro Fis release profile showed that after 24 h, 56% of Fis was released, and this value was increased to 73.6% after 6 days (Figure 12C). The encapsulation of Fis resulted in increasing the flavonoid cytotoxicity against CT26 cells (IC_50_ value of 8.0µg/mL) in comparison with free Fis (IC_50_ value of 28.5 µg/mL). Fis-loaded NMs were also injected in mice to test their effect on a subcutaneous CT26 carcinoma model developed in the animals, resulting in higher inhibition of tumor growth, malignant proliferation, and angiogenesis than free Fis (Figure 12D) [101].

PLA was copolymerized with D-α-tocopheryl polyethylene glycol 1000 succinate (TPGS) by ring-opening polymerization to develop Fis-loaded NMs investigated for their efficacy against MCF-7 cancer cells [102]. PLA-TPGS block copolymer NMs by nanoprecipitation presented an average diameter of 89 nm, an encapsulation efficiency higher than 90%, and a drug loading of 10.6%. They showed a sustained in vitro release, with 25% of encapsulated Fis released after 24 h and 80% after 96 h. The encapsulation determined an enhanced cytotoxic effect of Fis on MCF-7 cells in comparison with free Fis (IC_50_ values of 9.7 and 28.5 µg/mL, respectively). NMs antitumor efficacy was evaluated on an MCF-7 cancer cell-bearing tumor model, showing that free Fis significantly retarded the growth of the tumor; this effect was more pronounced for Fis-loaded NMs-treated mice groups [102].

Fis-loaded NPs made of a blend between PCL and PLGA-PEG-COOH were characterized for their in vitro release kinetics by simulating the gastrointestinal conditions [103]. The test revealed a slow Fis release at pH 2.0, indicating that the polymeric matrix was able to protect the drug inside the stomach in order to achieve most of the release in the intestinal tract. In particular, NPs containing higher amounts of PLGA-PEG-COOH showed a complete release after 24 h, while particles constituted by only PCL showed only a 70% cumulative release after 24 h. This was likely due to the presence of a more hydrophilic polymer favoring the diffusion of the medium inside the particles. The prepared particles showed the same antioxidant activity of free Fis and greater α-glucosidase (responsible for polysaccharide depolymerization) inhibitory activity than Acarbose, a widely used drug in the treatment of patients with type 2 diabetes [103].

Fis-loaded PLA NPs prepared by the spontaneous emulsion solvent diffusion method were investigated in vitro for the treatment of colon carcinoma cell line HCT116, as well as on the mouse 4T1 breast cancer xenograft tumor model [104]. The optimized particles presented an average diameter of 226.9 nm and encapsulation efficiency of 90.4%. NPs showed a slow and controlled Fis release in vitro (35% of loaded Fis was released within the first 24 h and 80% after 96 h), differently from the free drug, which was rapidly dissolved in the release medium (78% after 1 h). Encapsulated Fis presented a better antitumor effect against HCT116 cells (IC_50_ value of 29.3 µg/mL) than free Fis (IC_50_ value of 91.8 µg/mL). Pharmacokinetics of free and encapsulated drugs were evaluated after intravenous injection in Male Sprague-Dawley rats. Free Fis was swiftly eliminated from the plasma showing significantly lower half-life, mean residence and maximum concentration compared with those of encapsulated Fis. Fis-loaded NPs also presented an in vivo antitumor effect reducing the growth of the tumor in a more significant way in comparison to the free drug [104].

### 5.5. Icariin

Icariin (Ica): (3-((6-Deoxymannopyranosyl)oxy)-7-(glucopyranosyloxy)-5-hydroxy-2-(4-methoxyphenyl)-8-(3-methyl-2-butenyl)-4H-1-benzopyran-4-one) (Figure 8) is the most abundant flavonoid of the ancient Chinese medicinal plant *Epimedium prenylflavonoids* [105]. It has a variety of pharmacological activities such as antioxidant [106], anti-inflammatory [107], immunomodulation, antitumor [108], and neuroprotection [109].

Ica-loaded PEG-PLGA NMs with a size of 131.6 nm and encapsulation efficiency of 72.3% were prepared by a nanoprecipitation method [110]. In vitro release kinetics investigation at pH 6.8 showed an initial drug burst release phase (19.6% of loaded Ica released in 4 h) followed by a steady release phase (93.1% of loaded Ica released in 72 h). Enhanced cytotoxic effect on ASPC-1 cells was found for Ica-NMs in comparison with free drug [110].

Ica-loaded PHBV scaffolds for bone tissue engineering were fabricated by solvent casting combined with salt leaching, achieving a porosity of 88.8% and a pore size ranging between 50 and 200 µm [111]. In vitro investigation of drug release from PHBV scaffolds highlighted that after 24 h, Ica concentration was 4. 7 mg/L and that after 24 days, Ica concentration reached a plateau (16.4 mg/L). Ica-loading of scaffolds significantly increased the in vitro proliferation of human osteoblasts-like MG-63 cells in comparison to what was achieved with non-loaded PHBV scaffolds and Ica treatment of 2D culture on tissue culture polystyrene (TCPS) [111].

**Figure 13:**
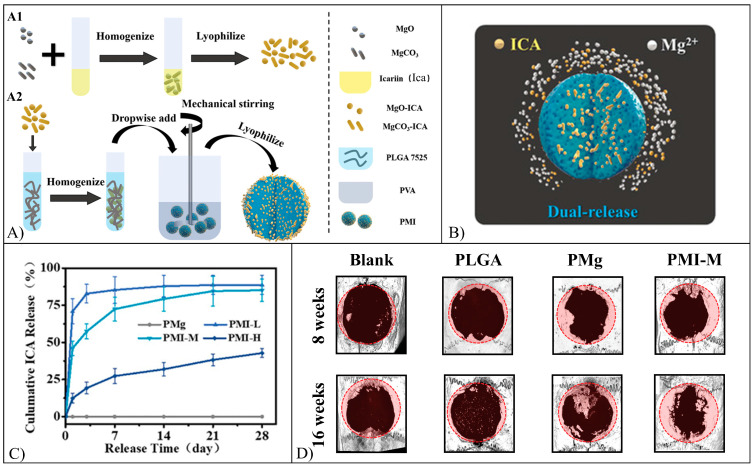
(**A**)Infographics of the fabrication of (**A1**) icariin (Ica)-loaded magnesium oxide (MgO)/magnesium carbonate (MgCO_3_) particles (MI) and corresponding (**A2**) MI-loaded poly(lactic-*co*-glycolic acid) (PLGA) (PMI) microspheres via emulsion technique employing poly(vinyl alcohol) (PVA) as a stabilizer; (**B**) schematic diagram presenting the dual-controlled release of magnesium ions (Mg^2+^)/Ica; (**C**) cumulative release concentrations of Ica form the microparticles loaded with different amounts of Ica: high (PMI-H), medium (PMI-M) and low (PMI-L), and (**D**) reconstructed 3D µ-CT images of rat cranium 8 and 16 weeks post-operation implanted with PLGA, PMg, and PMI-M microspheres using the unfilled defects as control. Reproduced with permission from [112], Wiley, 2020.

A bone tissue regeneration strategy involved Ica adsorption onto MgO/MgCO_3_ particles which were then encapsulated in PLGA microspheres employing an emulsion technique (Figure 13A) [112]. Different particles with an average diameter of 100–150 µm were synthesized by varying the amount of Ica loaded. In vitro dual release of Ica and Mg ions from the particles was detected (Figure 13B), and Ica release reached a steady phase only in the case of high drug loading, with a cumulative release of 42.8% after 28 days (Figure 13C).

In vitro investigations involving bone marrow mesenchymal stromal cells (BMSCs) demonstrated the osteoinductive effect of the adsorbed particles determining an increase of ALP activity and Col I content, as well as the formation of 3D microsphere-cells aggregates. In vivo bone regeneration investigation in Sprague-Dawley rat calvarial defects demonstrated that the new bone volume/total volume ratio and bone mineral density were larger in animals treated with loaded spheres in comparison to the case of non-loaded ones, as measured by means of Micro-CT (Figure 13D). Histological analysis also revealed loaded spheres with higher levels of collagen, osteopontin, and osteocalcin, as well as the formation of bone marrow-like structures [112].

**Figure 14:**
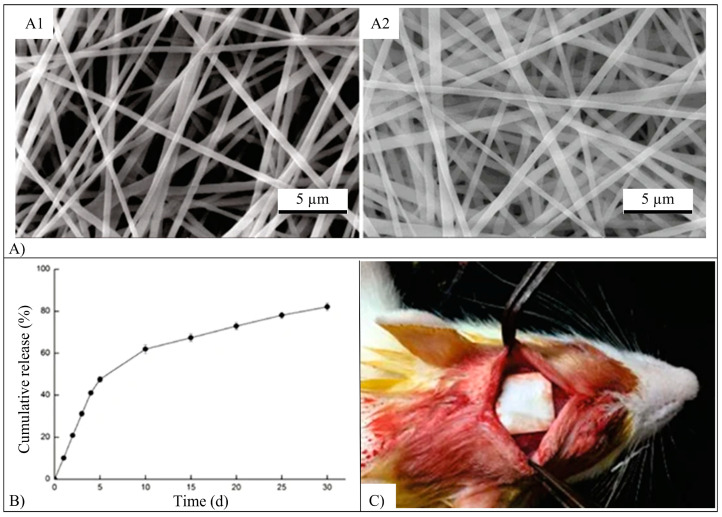
(**A**) SEM micrographs of (**A1**) Ica-loaded silk fibroin (SF)/poly(lactide-*co*-ε-caprolactone) (PLCL) scaffolds and (**A2**) SF/PLCL scaffolds; (**B**) cumulative release of Ica form the drug-loaded scaffolds; (**C**) implantation of the scaffold and coverage of the defect. Reproduced with permission from [113], Springer Nature, 2017.

Ica-loaded scaffolds made of poly(lactide-*co*-ε-caprolactone) (PLCL) and silk fibroin (SF) were fabricated employing coaxial electrospinning [113]. SEM micrograph (Figure 14A) analysis of the fabricated scaffolds showed uniformly distributed fibers with continuous, smooth, and homogeneous core-shell structures and an average diameter of 451.6 nm. In vitro, the Ica release profile was characterized by an initial burst release stage (47.5% of Ica released in 5 days) followed by a sustained release stage (82.1% Ica released after 30 days) (Figure 14B). The fabricated Ica-scaffolds were effective in promoting the in vitro BMSCs osteogenic differentiation and bone regeneration activity. Twelve weeks after implantation in critical-size rat calvarial defects (Figure 14C), the µ-CT analysis revealed that bone spread to cover most of the defect region, showing faster and greater bone formation in Ica-loaded scaffolds in comparison with the blank and negative control groups [113].

Core-shell scaffolds with an electrospun shell made of PCL/Col/Ha and a core made of Ica-loaded Cs and Col microspheres were investigated for bone regeneration [114]. The Cs microspheres (1 to 5 µm) were obtained by ionotropic gelation using tripolyphosphate (TPP) as an ionic crosslinker. In vitro, drug release studies showed that after 5 and 60 days, respectively, 30 and 70% of the total amount of Ica was released. The microspheres were added to a Col solution, and the obtained mixture was cast in a cylindrical mold and freeze-dried to obtain the core phase that was placed into the shell layer and cross-linked with genipin. The obtained scaffolds were implanted in tibial plateau bone defects in rabbits, and 12 weeks after implantation higher density of bone regeneration was observed in the case of Ica-loaded scaffolds [114].

Ica-loaded PLGA/tricalcium phosphate (TCP) composite scaffolds were fabricated employing a low-temperature 3D printing technique. Scaffolds loaded with three different amounts of Ica presented a well-interconnected macroporous structure (402 to 436 µm) and micropores (5 to 50 µm) on the pore wall surface [115]. In vitro release kinetics was characterized by a burst release and a sustained release phase up to 14 weeks, whose duration depended on Ica loading. MC3T3-E1 cells were cultured in vitro on the scaffolds, and Ica-loaded constructs showed superior cell proliferation and ALP concentration in comparison to non-loaded ones. In vivo activity of the scaffolds was evaluated through their implantation into the bone tunnel of the distal femora of rabbits affected by steroid-associated osteonecrosis (SAON), showing that Ica incorporation was effective in facilitating bone defect regeneration. New bone formed within the defect region after 8 weeks had higher mechanical strength and enhanced vascular remodeling in the case of Ica-loaded than in non-loaded scaffolds.

Small intestine submucosa (SIS) is an acellular collagenous extracellular matrix (ECM) material derived from the submucosal layer of the porcine intestine and approved by the FDA for tissue repair [116]. It contains growth factors controlling osteogenesis, bone tissue regeneration, and ECM formation. Ica-loading was achieved through immersion of an SIS scaffold into an ethanol solution of the flavonoid, followed by the evaporation of the solvent and rinsing of the sample in PBS. In vitro release of Ica was determined for scaffolds with different numbers of layers (1, 2, and 4). All scaffolds presented an initial burst release phase followed by a sustained release phase up to a cumulative variable value, depending on the number of layers and the amount of encapsulated Ica. 4-layers SIS scaffolds were employed for MC3T3 preosteoblast cell culture in vitro, showing that Ica loading did not affect cell viability over time. After implantation in a calvarial defect model in C57BL/6 mice, Ica-SIS scaffolds resulted in enhancing bone regeneration in a more significant way than non-loaded SIS scaffolds, leading to the formation of new tissue with bone marrow and blood vessels.

Embedding halloysite nanotubes (HNTs) loaded with Ica in thermosensitive Cs/glycerophosphate (GP) hydrogels were explored for the formation of an injectable system with an improved biological response and bone differentiation ability [117]. The thermosensitive hydrogel scaffolds were obtained by a sol-gel transition from hydrosol samples at 37 °C, employing either HNTs or nanotubes modified through surface functionalization with Cs (mHNTs). The encapsulation efficiency and Ica loading of HNTs were 10.0 and 78.2%, respectively, while for mHNTs were 95.2 and 12.5%. The gels resulted in being flowable at room temperature, but they became non-flowing at 37 °C. They displayed a 3D interconnected porous structure with an average pore size ranging from 139 to 151 µm. In vitro investigation demonstrated the cytocompatibility of the developed scaffolds, with no significant differences in terms of hASCs cell proliferation over time between non-loaded and Ica-loaded scaffolds. However, Ica-loaded scaffolds led to a significant increase in cell ALP activity, demonstrating the Ica activity in stimulating cell differentiation.

**Figure 15:**
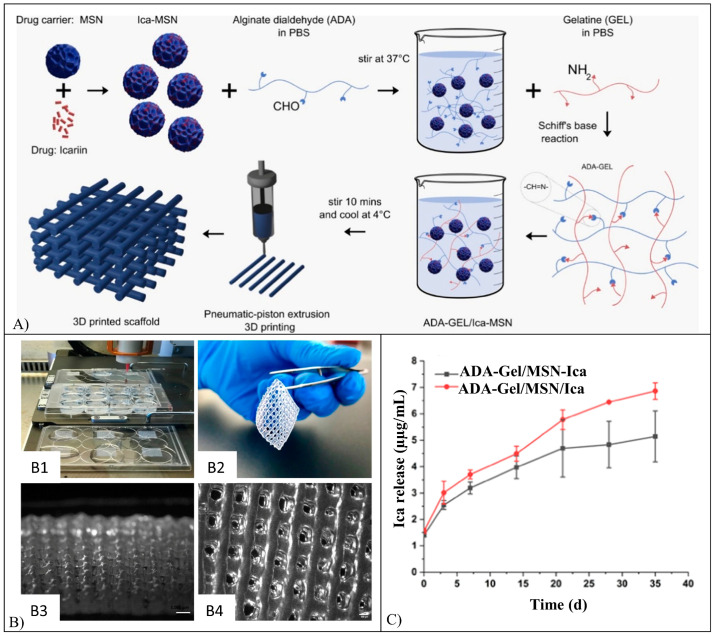
(**A**) Schematic illustration of the fabrication process of 3D printed icariin loaded ADA-Gel/MSN constructs; (**B**) (**B1**) 3D printing process, (**B2**) representative image of an ADA-Gel-based scaffold, (**B3**,**B4**) high magnification images of the constructs (scale bars represent 1000 and 500 μm respectively); (**C**) Ica release profile. Reproduced with permission from [118], Elsevier, 2021.

3D printed hydrogel nanocomposite scaffolds loaded with Ica were fabricated starting from mixing Gel and alginate di-aldehyde (ADA) in an aqueous solution, followed by the addition of mesoporous SiO_2_-CaO (MSN) NPs either loaded (ADA-Gel/MSN-Ica) or non-loaded with Ica [118]. In this second case, Ica was added to the Gel solution (ADA-Gel/MSN/Ica). By employing a pneumatic extrusion-based 3D printer, square shape, grid-like constructs were fabricated and then cross-linked in a CaCl_2_ solution (Figure 15A). The fabricated scaffolds presented a well-interconnected porous structure with an average pore dimension of 415 µm (Figure 15B). Both ADA-Gel/MSN/Ica and ADA-Gel/MSN-Ica scaffolds showed an initial burst release (respectively 1.6 and 1.4 µg/mL of Ica released after 1 h) followed by a sustained release over 35 days (Figure 15C). Moreover, in vitro cell culture investigations showed a progressive decrease of MC3T3 cell viability over time for ADA-Gel/MSN/Ica scaffolds, while in the case of ADA-Gel/MSN-Ica scaffolds, higher cell viability values were recorded. This could be due to the different drug release kinetics that, in the first case, can result in an Ica concentration in the culture medium higher than the toxic one [118].

Chondrocytes-laden Col hydrogels loaded with Ica were developed to investigate their role as an accelerator for chondrogenesis in cartilage tissue engineering [119]. The fabrication process involved the dissolution of Col in an acetic acid solution. After neutralization, Ica and chondrocytes were added to the solution, and the mixture was injected into a multi-well plate at 37 °C. After 28 days, 7.9% of Ica was still encapsulated in the hydrogels, and no differences in terms of distribution and survival of chondrocytes were detected between scaffolds loaded and non-loaded with Ica. The regenerative capability of the scaffolds was assessed through implantation in critical-size osteochondral defects created in mature New Zealand white rabbits. After 4 weeks, defects treated with Ica-loaded scaffolds resulted in being filled with a layer of opaque chondroid tissue with good integration with the host cartilage, while defects treated with scaffolds non-loaded with Ica were partially filled with opaque tissue, and most regions of the defect appeared as reddish myeloid tissue. After 12 weeks, all defects were completely filled by white neo-formed chondroid tissue, and their surfaces were nearly smooth. However, the surface of the defects treated with non-loaded scaffolds still had some small cracks.

In order to eliminate the initial burst release stage and obtain a gradual release for longer periods, Ica was covalently bonded to the polymeric scaffolding matrix [120,121]. A DMSO solution of Ica-methacrylate (Ica-MA) was mixed in PBS with hyaluronic acid-methacrylate (HA-MA) and a photoinitiator (Irgacure 2959). The obtained solution was added to a neutral Col aqueous solution, and the resulting mixture was cast in cylindrical molds and exposed to UV light. When Ica was simply blended with the hydrogel, a cumulative release of 70% was achieved within the first 24 h, while in the case of Ica covalently bonded to the scaffold, less than 20% of Ica was released after 24 h, and 75% of Ica was still encapsulated in the hydrogels after 96 h. This controlled release could be explained considering that rather than diffusion in the polymeric matrix, the kinetics followed the degradation of the scaffolds and the hydrolytic cleavage of the ester bonds between Ica and methacrylate [120]. Scaffolds covalently functionalized with Ica presented comparable chondrocyte cell viability, enhanced phenotype preservation, and increased synthesis of the cartilage-specific matrix in comparison to HA/Col scaffolds [121]. These kinds of hydrogels encapsulating BMMSCs were cultured in chondrogenic, osteogenic, and chondrogenic/osteogenic inductive media [122]. Ica-conjugated scaffolds determined higher cell expression of osteogenic and chondrogenic genes than HA/Col scaffolds, demonstrating that Ica maintained its pharmacological activity. In vivo investigations based on scaffolds implantation in rabbit osteochondral defects, demonstrated that Ica-HA/Col constructs not only promoted the regeneration of upper hyaline cartilage, but also enhanced the formation of new subchondral bone [122].

### 5.6. Icaritin

Icaritin (Ict): (3,5,7-trihydroxy-2-(4-methoxyphenyl)-8-(3-methylbut-2-enyl)chromen-4-one) (Figure 7) is a prenylflavonoid derivative form herb *Epimedii* exhibiting various pharmacological and biological activities, such as anticancer [123], anti-inflammatory [124], and antiosteoporosic activity [125].

An acid-base shift (ABS) method was developed to prepare Ict-loaded NMs based on Soluplus^®^/Poloxamer 407 [126]. For this purpose, an Ict solution in aqueous sodium hydroxide was dropped in a hydrochloric acid aqueous solution of the polymers obtaining NMs with an average diameter of 72.7 nm, encapsulation efficiency of 92.3% and drug loading of 13.2%. The in vitro release of Ict from the NMs was evaluated in simulated gastric fluid (SGF) and simulated intestinal fluid (SIF). NMs retained the drug more in the gastric than in the intestinal environment (after 24 h, about 19.0% and 77.3% of Ict were released in SGF and SIF, respectively). Bioavailability investigation in a beagle dog model showed that plasma concentration and total cumulative release for Ict-NMs were 9.5- and 14.9-fold higher than those of reference oil suspension. The mechanism of transcellular transport of Ict-NMs was determined employing a Caco-2 cell monolayer reproducing the intestinal epithelium, finding that the NMs were endocytosed by epithelial cells and exocytosed in the form of free Ict [126].

PLGA-PEG-aminoethyl anisamide NPs dually loaded with Ict and Dox were synthesized employing a nanoprecipitation method and investigated for liver cancer treatment [127]. Their average particle size was 138 nm, and Ict encapsulation efficiency resulted in the range between 94.1 and 96.7%, depending on the molar ratio between Ict and Dox. Cumulative drug release became pH-responsive, increasing from 35% at pH 7.4 to 80% at pH 5.5. Pharmacokinetics parameters after injection through the mouse tail vein demonstrated that the encapsulation of the drugs resulted in higher plasma concentration in comparison to the case of free drugs. Ict and Dox remodeled the immunosuppressive microenvironment and triggered a robust immunosuppressive memory response, exerting a satisfactory anti-HCC effect at an early and advanced stage [127].

Ict-loaded PLGA/TCP composite scaffolds were fabricated employing a low-temperature 3D Printing technique which involved the addition of PLGA, TCP, and Ict to 1,4-dioxane. The resulting paste was extruded by a computer-driven nozzle at −28 °C to form homogeneous cubic porous scaffolds with interconnected macropores ranging from 300 to 450 µm [128,129]. In vitro studies demonstrated that Ict was released during scaffold degradation and that the cumulative drug release after 12 weeks was 72%. Loading the scaffolds with Ict promoted the growth in vitro of BMMSCs as well as their differentiation into osteoblasts. Moreover, the osteogenic and angiogenic effect of the Ict scaffolds was evaluated in vivo employing rabbits with a transverse coronal bone tunnel. The implantation site healed without any sing of infection or inflammation after 12 weeks, with an osteopromotive effect and enhanced neovascularization in the case of Ict-loaded scaffolds [129]. Ica-loaded PLGA/TCP scaffolds were also tested for large bone defect repair in the adult emu, and they effectively promoted bone defect repair and prevented hip joint collapse [130].

### 5.7. Morin

Morin (Mor): (2-(2,4-Dihydroxyphenyl)-3,5,7-trihydroxy-4H-chromene-4-one) (Figure 7) is a flavonol found in various plants, particularly the *Moraceae* family [131]. It presents antiinflammatory, antioxidant, neuroprotective [132], antifungal, and anticancer activities [133].

Mor-hydrate (MH)-loaded simple NPs (MH-NPs) and NPs based on different mixed polymers (MH-mNPs), i.e., hyaluronic acid-poly(butyl cyanoacrylate) (HA-PBCA) block copolymer and D-alpha-tocopheryl polyethylene glycol 1000 succinate (TPGS) which is a water-soluble derivative of vitamin E were prepared by a probe-type ultrasonic and dialysis method achieving a mean diameter of 134.7 nm [134]. MH encapsulation resulted in higher in vitro cytotoxicity towards the A549 lung adenocarcinoma cell line compared with free MH. Moreover, MH-mNPs showed a greater cytotoxic effect compared to NPs not containing TPGS (MH-NPs), probably because of the synergistic antitumor activity of TPGS in the presence of the flavonoid caused by TPGS’s ability to induce ROS-generation and cancer cells apoptosis, as well as by the combination of MH and TPGS ability to inhibit P-glycoprotein (P-gp) expressed in the cells, thus reducing drug efflux. mNPs cellular uptake was also increased by the polyelectrolyte interactions between the carboxylate groups of HA, which are distributed on the surface of the particles, and the CD44 receptors.

The endocytosis kinetics study, monitored by fluorescence microscopy and confocal laser scanning microscopy (CLSM) entrapping a fluorescent molecule (C6) in the NPs, revealed that the NPs intracellular uptake increased in a time-dependent manner, with a higher intracellular uptake for MH-mNPs. The increased tumor inhibitory effect of MH-mNPs was also demonstrated in vivo, highlighting the prolonged survival time of the treated mice, with survival rates at day 20 of 57% for MH-NPs and 71% for MH-mNPs [134].

**Figure 16:**
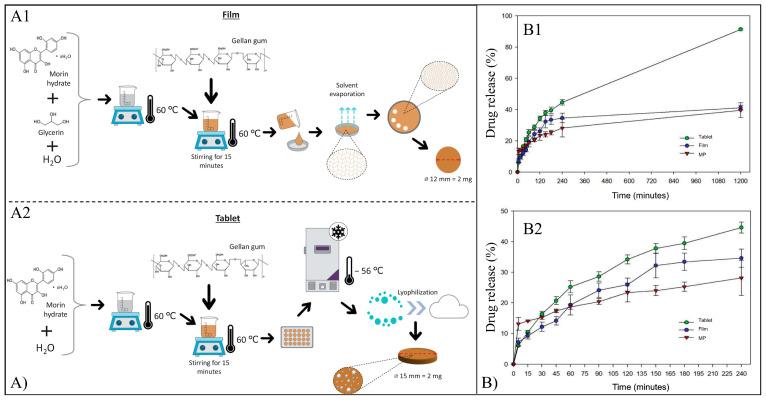
(**A**) schematic representation of the process for the development of morin hydrate loaded gellan gum/alginate films (**A1**) and tablets (**A2**); (**B**) drug release diagram of morin release profile from tablets, films, and microparticles (MP) (**B1**) for all experimental time and (**B2**) zoom on the first 4 h of the experiment. Reproduced with permission from [135], Elsevier, 2020.

Mor-loaded microparticles (MPs), films, and tablets made of alginate and gellan gum were obtained by ionotropic gelation, solvent casting, and lyophilization, respectively (Figure 16A), and investigated as antimicrobial devices [135]. In vitro drug release (Figure 16B) investigation showed that after 20 h, the tablets, films and MPs reached an average cumulative release percentage of 91, 41, and 60%, respectively. The higher percentage of the release of tablets was likely related to their porous structure formed during lyophilization. The antibacterial activity of the systems was evaluated on *S. mutans,* and it was shown that films and tablets affected bacterial viability more markedly than MPs. The influence of films and tablets on the establishment of monospecies biofilms of *A. naeslundii* and *S. mutans* was evaluated, demonstrating that the tablets reduced *A. naeslundii* viability in a more significant way than the films.

### 5.8. Myricetin

Myricetin (Myr): (3,5,7-Trihydroxy-2-(3,4,5-trihydroxyphenyl)-4H-chromen-4-one) (Figure 7) is a flavonoid compound, mainly found in vegetables, fruits, nuts, berries, herbs, and plants, as well as in beverages, such as tea and wine [73]. It is characterized by many pharmacological properties, such as antibacterial, antioxidant, antidiabetic, and immunomodulatory properties [73].

NPs with a diameter ranging from 12 to 47 nm and constituted of a poly-(dimethylaminoethylmethacrylate)-b-poly-(dimethylaminoethyl methacrylate-co-butyl methacrylate-co-propylacrylic acid) core and a poly(dimethylaminoethylmethacrylate) corona were loaded with Myr via electrostatic interactions with the cationic corona [136]. The corona electrostatic interactions did not affect the loading or the release of farnesol, a hydrophobic drug loaded in the hydrophobic core.

Eudragit RS100^®^ was employed to produce nanoprecipitation NCs loaded with dihydromyricetin (dMyr), achieving an encapsulation efficiency of 80.9% [137]. The NCs showed in vitro release kinetics characterized by two phases, i.e., an initial burst release phase and a second sustained release phase. dMyr-loaded NCs demonstrated antimicrobial properties and the ability to eradicate the biofilm population, making them effective against catheter-related urinary tract infections.

Myr was encapsulated into Cs functionalized Pluronic P123/F68 NMs and prepared to employ a thin film hydration method in order to enhance its activity in the treatment of brain cancer [138]. The average NMs diameter was 51.5 nm, while Myr loading and encapsulation efficiency were reported to be 15.6% and 91.7%, respectively. The NMs released Myr in vitro in a cumulative manner with time, with more than 90% of the loaded drug released in the first 12 h. Finally, it resulted that Myr-loaded NMs achieved higher brain accumulation and enhanced brain penetration efficiency after intragastric administration in mice, compared with free Myr in solution at the same dose.

### 5.9. Naringenin

Naringenin (Nag) (5,7-dihydroxy-2-(4-hydroxyphenyl)-2,3-dihydrochromen-4-one) (Figure 7) is a flavanone commonly found in citrus fruits such as orange, lemon, and lime [139]. It has a wide spectrum of biological activities, such as antidiabetic, anticancer, hepatoprotective, neuroprotective, cardioprotective, nephroprotective, gastroprotective, and antimicrobial activity [140].

Nag was encapsulated in a wide variety of polymeric NPs employing different synthesis techniques. For instance, Nag-loaded PLGA NPs were prepared by employing a nanoprecipitation method to verify their therapeutic potential against autism spectrum disorders [141]. The encapsulation increased the therapeutic efficacy of Nag in comparison with its free form, and coating the NPs with reduced glutathione and Tween 80 further increased drug bioavailability and brain uptake. Another study involved the employment of an emulsion-diffusion-evaporation method to prepare PLGA NPs loaded with Nag for the treatment of diabetes mellitus [142]. Particle size results were 129 nm with 70% of Nag encapsulation efficiency. In vitro release kinetics of Nag from the NPs results were slower and more controlled in comparison with the dissolution of free Nag (after 4 h, 12.4 µM of Nag was released from the NPs while 51.3 µM of the free drug was dissolved in the release medium). In vivo tests carried out on diabetic rats demonstrated that Nag-loaded PLGA NPs presented an enhanced effect than free Nag in ameliorating diabetogenic effects, such as hyperglycemia and hyperlipidemia [142].

**Figure 17:**
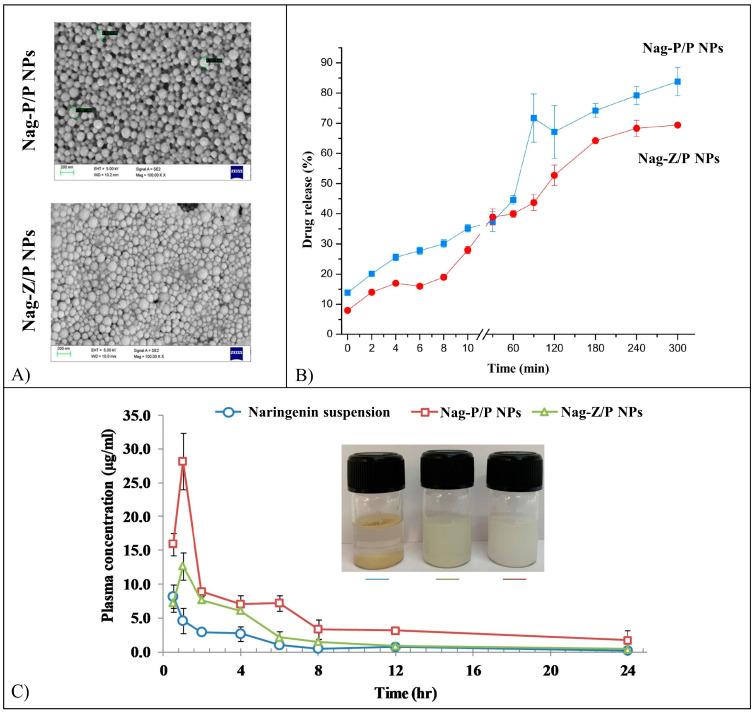
(**A**) SEM image of Nag-P/P and Nag-Z/P NPs; (**B**) release profile of Nag-loaded nanoformulations at different digestion phases (simulated salivary fluid (SSF): 0–10 min; simulated gastric fluid (SGF): 10–120 min; simulated intestinal fluid (SIF): 120–300 min; (**C**) plasma concentration-time profile for the different Nag-loaded formulations. Reproduced with permission from [143], Elsevier, 2022.

Nag-loaded PLA/PVA NPs (Nag-P/P NPs) with an average size from 147 to 409 nm and encapsulation efficiency from 22.1 to 80.4%, depending on the percentage of the three compounds, were prepared by solvent evaporation method [143]. Nag-loaded Zein/pectin NPs (Nag-Z/P) with a mean particle size from 185 to 403 nm and encapsulation efficiency from 19.5 to 68.9%, depending on the pectin concentration and pH of the system, was prepared through anti-solvent precipitation and electrostatic deposition method. Further characterization was carried out on Nag-P/P NPs with a size of 170 nm (Figure 17A) and encapsulation efficiency of 79.3%, as well as on Nag-Z/P NPs with a size of 220 nm (Figure 17A) and an encapsulation efficiency of 62.5%. The antioxidant activity of the nano-formulations resulted in being concentration-dependent, increasing from 21 to 95% and from 34 to 99% for Nag-P/P and Nag-Z/P NPs, respectively, over the concentration range of 10–180 mg/L. The radical scavenging activity of Nag increased after the encapsulation process, likely because of the improved dissolution property of Nag in an aqueous medium. In vitro release of Nag from both formulations was measured in simulated salivary fluid (SSF), SGF, and SIF (Figure 17B). Nag release in SSF was monitored for 10 min, resulting in a final cumulative release of 10–20% for both formulations. Nag release in SGF was studied for 2 h resulting in a final cumulative release of 21 and 32% from Nag-P/P and Nag-Z/P NPs, respectively. Finally, after 3 h of incubation in SIF Nag-P/P NPs reached a cumulative drug release of 88%, while Nag-Z/P NPs reached a cumulative drug release of 71%. 

Nag in vivo bioavailability was determined by analyzing its plasma concentration after oral administration of the formulations in rats (Figure 17C). The maximum plasma concentration and its relative bioavailability were higher for NPs treated rats in comparison with free Nag-treated rats. Nag-P/P NPs showed a higher relative bioavailability in comparison with Nag-Z/P NPs, likely because of the increased mucoadhesive properties of the second one [143].

Cationic-polymeric NPs based on Eudragit E100^®^, a copolymer constituted by a 1:2:1 ratio of methyl methacrylate, N, N-dimethylamino ethyl methacrylate, and butyl methacrylate monomers, were employed for the encapsulation of Nag through an emulsion-diffusion-evaporation method and investigated for the treatment of colorectal cancer [144]. Varying the concentration of the stabilizing agent (Poloxamer 188) particle mean diameter ranged from 430.4 to 798.1 nm, while Nag encapsulation efficiency and loading varied from 55.5 to 74.7% and from 0.4 to 1.3%, respectively. In vitro Nag release from the particles was characterized by an initial burst release phase within the first 30 min followed by a sustained diffusion-controlled release phase, resulting after 24 h in more than 75% of cumulative Nag release. Pharmacokinetics parameters showed a significant improvement of Nag bioavailability after encapsulation in Eudragit^®^ NPs in comparison with free Nag, as well as a higher suppression of tumor growth.

Core-shell Cs NPs coated with sodium alginate (Alg) and loaded with Nag were prepared by dropping a sodium sulfate solution in a Cs/Nag solution under sonication and then dropping the resulting dispersion in an Alg solution [145]. The hydrodynamic diameter of the NPs ranged from 150 to 300 nm, Nag encapsulation efficiency between 57 and 98%, and Nag loading between 7.4 and 19.9%. Nag release from NPs was delayed at pH 1.2 because the outer layer of Alg did not swell due to the presence of undissociated carboxyl groups, as well as to the formation of hydrogen bonds within the carboxyl and hydroxyl groups of alginate. At pH 7.4, more than 90% of Nag was released, likely because of the repulsion between carboxylate ions of alginate that aided the penetration of the solvent in the core of the particles. Ex vivo and in vivo mucoadhesion studies showed that the particles firmly attached to the lumen of rat intestine, allowing a sustained release and the permeation of Nag from intestinal microvilli to the circulatory system. The prepared NPs presented an anti-diabetic effect. Normalization of blood glucose was achieved after 27 days of treatment of NPs in diabetic rats. Histology of the pancreas confirmed the antidiabetic activity of the particles revealing a significant improvement and regeneration in the structural complexity of the Langerhans cells as compared to diabetic control. Hepatic sections showed that core-shell NPs caused the revival of the hepatic tissue architecture.

Nag-loaded sulfobutylether-β-cyclodextrin/chitosan (SCd/Cs) NPs were prepared by ionic gelation and investigated for ophthalmic administration [146]. The NPs presented a mean diameter of 190 nm and a Nag encapsulation efficiency of 67.1%. In simulated tear fluid, Nag-loaded SCd/Cs NPs showed a more controlled and slower release profile in comparison with Nag-SCd because of the impediment of the Cs matrix but a faster release than free Nag because of its low solubility. Investigations using a rabbit model demonstrated that the formulation could be considered safe for ocular use and that the encapsulation of Nag into NPs increased the bioavailability of the drug and prolonged its residence time [146]. 

Nag-polyvinylpyrrolidone (PVP) self-assembled nanocomplexes (NXs) were prepared employing a thin-film hydration method and investigated for ocular delivery [147]. The optimized formulation presented NXs size of 6.7 nm and a Nag complexation efficiency of 98.5%. The complexation of Nag increased its water solubility from 40.2 µg/mL to 37.0 mg/mL. Nag-PVP NXs exhibited a concentration and time-dependent antioxidant activity, which could be optimized at higher levels than that of free Nag. No sign of tissue damage or clinical abnormalities in the conjunctiva, cornea, iris, or pupil was found in a rabbit model after Nag-loaded NXs administration. Moreover, it was demonstrated that Nag-loaded NXs presented a stronger anti-inflammatory effect compared to free Nag and DIC eyes drop, a widely used nonsteroidal anti-inflammation eye drop [147].

### 5.10. Naringin

Naringin (Nar): (5-Hydroxy-2-(4-hydroxyphenyl)-7-(2-O-alpha-L-rhamnopyranosyl-beta-D-glucopyranosyloxy)-4-chromanon) (Figure 8) a glycosylated flavanone mainly found in citrus fruits such as mandarin (*Citrus reticulata*), pummelo (*Citrus maxima*) and sweet orange (*Citrus sinensis*) [148]. It has a wide range of biological activities, including anti-inflammatory, anticancer, antioxidant, antigenotoxic, neuroprotective, and osteoconductive activity [149].

To increase their stability in the gastric environment, Nar and Nag were encapsulated into cellulose acetate phthalate (CAP) MPs, with a mean diameter ranging between 1 and 6 µm, obtained by spray drying. Drug loading ranged from 10 to 31% for Nar-loaded MPs and from 13 to 40% for Nag-loaded MPs [150]. Nar-loaded MPs released only 23% of the loaded drug in the gastric medium, while 84% of the free drug dissolved within the same conditions. In simulated intestinal fluid (SIF), 85–90% of the encapsulated drug was released within 60 min. Nag-loaded MPs released only 9–12% of the loaded drug in the gastric medium and about 40 to 50% in SIF demonstrating the ability of the particles to stabilize the drugs in the gastric environment and release them in the intestinal environment [150].

Nar-loaded PLGA NPs were prepared using a solvent-evaporation emulsification technique with an optimized combination of stabilizers (Pluronic-F68, sodium deoxycholate, and PVA) [151]. These particles presented a mean diameter of 179.7 nm with a drug encapsulation efficiency of 74%. In vitro cumulative Nar release was 82% after 24 h of incubation. In vivo assessment of chronic arthritis reduction in rat models demonstrated that Nar-loaded NPs have a greater anti-inflammatory and antioxidant effect than free Nag [151].

### 5.11. Oroxylin A

Oroxylin A (OrA): (5,7-dihydroxy-6-methoxy-2-phenylchromen-4-one) (Figure 7) is a flavonoid found in *Scutellariae radix* that has a wide range of biological activities, such as anti-inflammatory, anticancer, and neurofunction improvement activity [152].

OrA-loaded PDots, aggregation-induced light emission-based drug delivery systems with a diameter of about 50 nm were prepared by the nanoprecipitation method, exploiting the aggregation of conjugated polymers attributed to a sharp enhancement in the solvent polarity [153]. The conjugated polymer was dissolved in THF together with OrA, and the solution was dropped in a water bath. The synthesized particles were characterized by a drug loading of 16.0% and slower release kinetics in comparison with free drugs. The cellular uptake of the particles was evaluated by employing A431 cells. Fluorescence signals inside the cells correlated to the PDots were observed after 4 h of incubation. Moreover, OrA-loaded PDots exhibited significant cytotoxicity against A431 cells after 48 h. Upon injection in an A431 tumor-bearing mice model, OrA-loaded PDots exerted a strong preference for accumulating in the tumor tissue after 6 h. After 48 h from the injection, ex vivo imaging confirmed that PDots preferentially targeted tumor tissue more than other organs. After 18 days of treatment, tumor growth in an OrA-loaded PDots-treated mice model was suppressed, confirming their anticancer activity [153]. 

### 5.12. Phloretin

Phloretin (Phl) (3-(4-Hydroxyphenyl)-1-(2,4,6-trihydroxyphenyl)propan-1-one) (Figure 7) is a flavonoid mainly found in apple (*Malus domestica*), pear (*Pyrus amygdaliformis*), and strawberry fruit (*Fragaria x ananassa*) [154]. It is a dihydrochalcone with antioxidant, anti-inflammatory, antidiabetic, anticancer, hepatoprotective, cardiovascular protective, and neuroprotective activities [155].

Phl-loaded Cs NPs with a mean diameter of 80.2 nm were synthesized through an ionic gelation method [156]. In vitro drug release demonstrated that after 48 h of incubation, 21% of encapsulated Phl was released at pH 7.4, whereas 71.6% of encapsulated Phl was released at pH 5.4. The obtained particles showed cytotoxic effects towards oral cancer cells (KB cell line) with an IC_50_ value of 20.3 µg/mL but not towards non-cancerous human cells (HEK-293 cell line) even at higher concentrations. The progressive morphological changes of Phl-Cs NPs-treated KB cells were observed and, differently from untreated cells, which showed normal nuclei, normal organelle, and normal membrane, they showed apoptotic morphological changes [156].

PCL, copaiba oil, sorbitan monostearate, and Phl were dissolved in acetone, the organic solution was dropped into an aqueous solution of polysorbate 80, and the organic solvent was evaporated to prepare Phl-loaded NCs with an average diameter of 252 nm [157]. After 4 h, the NCs released 20% of the encapsulated Phl, while 40% of free Phl diffused through the dialysis bag, confirming the control of drug release kinetics provided by the polymeric matrix. The release kinetics showed an initial burst release phase followed by a steady release phase. Phl-NCs were tested on human melanoma cells (SKMEL-28 cell line), as well as on human fibroblasts (MRC5) and human keratinocytes (HACAT). Lower proliferation rate was observed in the presence of Phl-loaded NCS for melanoma cells, while no significant effect was observed for fibroblasts and keratinocytes. Lecigel^®^, a gelling agent with emulsifying properties composed of sodium acrylate copolymers and lecithin, was chosen to produce hydrogels containing the loaded NCs (HG-Phl-NCs). A porcine skin permeation/penetration test was carried out employing either the suspension or the hydrogel. While Phl was not able to penetrate through the layers of the skin, the developed formulations presented different profiles of penetration in skin layers. Phl-NCs and HG-Phl-NCs resulted in an enhanced reservoir effect on the stratum corneum, with HG-Phl-NCs leading to a high Phl concentration in the epidermis [157].

### 5.13. Quercetin

Quercetin (Que) (2-(3,4-dihydroxyphenyl)-3,5,7-trihydroxy-4H-1-benzopyran-4-one) (Figure 7) is a naturally occurring flavonol distributed in many edible plants, such as fruits (mainly citrus), green leafy vegetables, as well as many seeds, buckwheat, nuts, flowers, barks, broccoli, olive oil, apples, onions, green tea, red grapes, red wine, dark cherries, and berries, such as blueberries and cranberries [154]. It possesses a great range of therapeutic properties, such as antioxidant, antibacterial, hepatoprotective, anticancer, antidiabetic, and anti-inflammatory activity [158].

PLGA NPs by nanoprecipitation was first employed for the encapsulation of Que [159]. The obtained NPs’ diameter and encapsulation efficiency ranged between 130 and 180 nm and between 20 and 40%, respectively. The in vitro release profile revealed that after 24 h, only 38.7% of Que was released from the NPs. Nevertheless, encapsulation resulted in increasing Que cytotoxic activity against the A549 cancer cell line in comparison with free Que [159]. Different other techniques were evaluated for the production of NPs loaded with Que. For instance, electrospraying was successfully employed for the fabrication of Que-loaded PLGA particles with a mean diameter of 384.9 nm [160]. In vitro degradation investigation highlighted that blank PLGA NPs in PBS solution lost their shape and agglomerated after 1 day, then created a film, and were almost completely degraded after 20 days. Que-loaded NPs instead presented a slower degradation process since they lost their shape and started to agglomerate after 5 days and needed 50 days to completely degrade. The in vitro release behavior of Que-loaded NPs followed a biphasic model with a first burst release phase during which 27% of loaded Que was released and then a sustained release phase with a total cumulative Que release of 96% after 50 days [160]. A single emulsion-solvent evaporation technique was also employed for the preparation of Que-loaded PLGA NPs with a diameter ranging from 118 to 279 nm and an encapsulation efficiency ranging from 73.6 to 86.5%, depending on formulation conditions [161]. The two optimized formulations released in vitro 56 and 65% of the loaded Que after 24 h, and they presented a maximum antioxidant activity of 80%. Moreover, after oral administration of Que-PLGA NPs, rats presented a significant increase in diuretic action and activity in comparison with the control group and free Que-treated rats. Another study reported the preparation of Que-loaded PLGA NPs presenting a mean diameter ranging from 100 to 150 nm by means of a double emulsion-solvent evaporation method to study their effect on Alzheimer’s disease treatment [162]. Indeed, Que is able to attenuate the toxic effect of the amyloid-β peptides (Aβ) and reduce their tendency to aggregate and form fibrils. In addition, it strongly chelates metal cations that accumulate in the Aβ aggregates, reducing the harmful effect of Alzheimer disease. In comparison to free Que, Que-PLGA NPs showed increased efficiency in inhibiting Aβ_42_ peptides aggregate fibril formation, as well as in dissolving the formed aggregates. The release kinetics of Que-PLGA NPs showed an initial burst release phase, followed by a sustained release phase over the entire time of the experiment, while total solubilization of free Que was reached after 2 h. In vitro cytotoxic assay carried out on SH-SY5Y cells demonstrated that Que-loaded PLGA NPs were able to partially block the Zn^2+^/Aβ_42_ system-induced cytotoxicity. In vivo test conducted on a rat model showed that the treatment with Que-loaded PLGA NPs improved their spatial memory in a dose dependent manner and attenuated the impairment of the non-spatial working memory [162].

Among its many bioactive properties, Que has the capability to inhibit P-glycoprotein (P-gp) receptors causing the efflux from the intestinal lumen and a consequent low oral bioavailability of etoposide, a polophytotoxin derivative promoting the apoptosis of cancer cells. In this context, PLGA NPs dual-loaded with Que and etoposide were developed using a single emulsification (o/w) solvent evaporation technique [163]. The dual-loaded particles released 65% of encapsulated Que after 24 h and 65% of etoposide after 48 h. This release pattern is of particular interest because it favors Que’s interaction with the P-gp receptors before that of etoposide, which is a podophyllotoxin derivative and acts as a topoisomerase II inhibitor promoting apoptosis of the cancer cells. Pharmacokinetics profiles after oral administration in Wistar rats revealed that dual-loaded NPs enabled the achievement of the highest etoposide concentration in the plasma in comparison with etoposide-loaded NPs and free etoposide.

Que also demonstrated the ability to increase the bioactivity of NPs prepared by a w/o/w emulsion method and made of a composite between superparamagnetic silica NPs (SiN) and PLGA [164]. In vitro release investigation demonstrated that Que was released faster in the case of SiN/PLGA composite NPs than in the case of PLGA NPs, with a cumulative release after 80 h of 90% and 55%, respectively. The developed Que-loaded composite NPs were demonstrated to support the in vitro adhesion and proliferation of H9c2 heart cells, as well as their expression of cardiac proteins.

PCL NPs loaded with Que were successfully prepared by employing a nanoprecipitation method [165]. The mean particle size ranged from 162 to 259 nm, and the encapsulation efficiency was from 36.5 to 78.1%. The cumulative percentage release at 48 h ranged from 55.4 to 89.3%. Que release profile presented an initial burst release phase within the first 10 h, followed by a sustained release phase until 48 h [165].

Analogous to what was previously described for PLA, copolymerization of PCL with TPGS is a promising approach to increase polymeric matrix hydrophilicity and compatibility with blood [166]. PCL-TPGS copolymers were synthesized through ring-opening polymerization of ε-caprolactone (ε-CL) using TPGS as the initiator. By varying the comonomers molar ratio in the reaction mixture, PCL and TPGS in the synthesized copolymers resulted in being in the range between 25:75 to 67:33. The copolymers were employed for the preparation of Que-loaded NPs through a nanoprecipitation method for cancer treatment. The variation of the hydrophilic/hydrophobic balance affected particle size, Que loading, and Que release kinetics due to an effect on NPs stability in an aqueous medium as well as on the compatibility between polymer and Que. All particles showed a sustained release over 24 h with no initial burst release; the faster release rate and the higher cumulative amount of released Que were reached for the smallest PCL to TPGS ratio. In vitro studies demonstrated that the encapsulation of Que into PCL-TPGS NPs determined a reduction of the IC_50_ value of 2.9–4.6 folds as compared to free drug solution, determining an increase of its cytotoxic effect against the SKBR3 breast cancer cell line [166].

Two optimized formulations of Que-loaded bovine serum albumin (BSA) NPs with a size of 152 and 132 nm were prepared by a desolvation technique [167]. The NPs presented a slow drug release with second-order kinetics, characterized by a first rapid-release phase followed by a second slow-release phase. The particles were able to scavenge 2,2′-azino-bis(3-ethylbenzothiazoline-6-sulfonic acid) (ABTS•+) and hypochlorous acid, which is produced by organisms through oxidation of Cl^-^ ions at sites of inflammation, demonstrating their antioxidant activity even if it was lower to that one associated with pure Que. *Dolichos biflorus* L. seeds extracted Que was loaded in BSA-coated poly(propylene glycol)-block-poly(ethylene glycol)-block-poly(propylene glycol) (PPG-PEG-PPG) NPs for application in inflammation modulation [168]. The particles prepared by the dialysis method had a diameter that ranged from 322 to 840 nm, depending on the Que/polymer ratio. The BSA/polyether system provided a slow and gradual release of Que (about 60% after 72 h), effective in reducing sodium oxalate-induced cytotoxicity on Maldin-Darby canine kidney epithelial cells [168].

Cs NPs prepared by an ionic gelation method were employed for the encapsulation of Que [169,170]. The preparation involved dropping a TPP/Que solution in a Cs solution to obtain NPs with tunable size depending on Cs to TPP ratio. The NPs had encapsulation efficiency values of around 80% and an in vitro release profile presenting a biphasic nature, with a first phase characterized by the burst release of the Que adsorbed to the particle surface and a second phase characterized by the sustained release of the entrapped drug. Encapsulated Que anticancer potential was evaluated through tumor xenograft studies in C57BL6 mice using A549 and MDA-MB-468 cells. After 5 weeks of treatment, tumor volume and the excised tumor weight in both groups of tumor-bearing mice were reduced not only in comparison with the control groups but also with the groups treated with free Que [169]. Que was also encapsulated into pH-sensitive N-succinyl chitosan (NCS)/Alginate (Alg) NPs reaching an encapsulation efficiency of 94% [171]. The release kinetics was studied by reproducing the pH values of the gastrointestinal tract, demonstrating that at pH 1.2 (corresponding to the pH of the stomach in the presence of food), all samples retained most of the drug (only 24–36% of Que was released from NCS/Alg NPs) probably due to hydrogen bond interactions between Alg and NCS which reduce the water penetration and swelling of the particles. At a pH of 7.4 (that represents the pH in the intestinal tract), a sustained drug release was observed together with the progressive swelling of the particles until their breaking and complete drug release after 6 h of incubation. A pronounced oral hypoglycemic effect of Que-loaded NPs, without systemic toxicity, was demonstrated in a diabetic rat model in comparison to the case of free Que administration [171].

**Figure 18:**
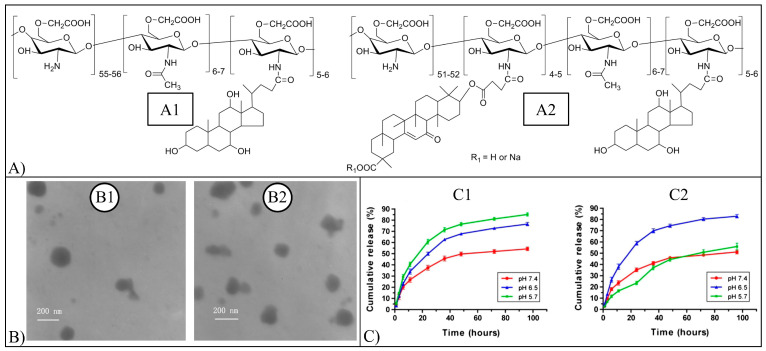
(**A**) Chemical structures of (**A1**) O-carboxymethyl chitosan modified with cholic acid (CMCA), and (**A2**) CMCA conjugated with glycyrrhetinic acid (GA-CMCA); (**B**) TEM micrographs of (**B1**) quercetin-loaded CMCA self aggregates, and (**B2**) quercetin-loaded GA-CMCA self aggregates; (**C**) in vitro release of quercetin from (**C1**) CMCA and (**C2**) QC-GA-CMCA systems at different pHs. Reproduced with permission from [172], Elsevier, 2015.

O-carboxymethylated chitosan (OCMC) is a water-soluble chitosan derivative synthesized by a substitution reaction of chitosan and monochloroacetic acid to achieve increased stability and bioactivity [172]. OCMC was modified with cholic acid (CMCA) (Figure 18A) to prepare novel self-aggregates exploitable for Que encapsulation. Two types of Que self-aggregates were prepared by sonication and dialysis, one made of Que and CMCA, and the second one obtained by conjugation of glycyrrhetinic acid (GA), a ligand for liver targeting, to CMCA. Increasing the drug-to-carrier feed ratio in optimized ranges, particle size, and encapsulation efficiency increased, with optimized values for Que-GA-CMCA of 185.5 nm and 56.9% and for Que-CMCA of 211.4 nm and 47.0%, respectively (Figure 18B). Both systems presented sustained and pH-dependent drug release kinetics constituted by an initial burst release phase followed by a sustained release phase (Figure 18C). Que-CMCA system released 85, 75, and 55% of Que after 96 h at pH of 5.7, 6.5, and 7.4, respectively, because of an increased protonation degree of the carboxymethyl groups causing the disassembly of self-aggregates. Que-GA-CMCA system released 80 and 50% of the loaded drug after 96 h at pH of 6.5 and 7.4, respectively. At a pH of 5.7, the aggregation and precipitation of self-aggregates were observed, resulting in a diffusion-mediate delayed drug release without any burst release. This phenomenon could be attributed to the protonation of the sodium salt GA on the self-aggregates surface. Que-GA-CMCA showed the highest cytotoxicity towards HepG2 cells with an IC_50_ of 12.5 µg/mL, while Que-CMCA and Que presented an IC_50_ of 16.2 and 17.2 µg/mL, respectively. This result depends on the presence of GA modification, which can induce receptor-mediated internalization and increase the accumulation of Que inside cells. Moreover, cells treated with Que and Que-loaded self-aggregates exhibited chromatin condensation or nuclear fragmentation, which are morphological changes characterizing cell apoptosis. Studies based on injection of Que-CMCA and Que-GA-CMCA in male adult rats demonstrated that after 1 h, Que concentration in blood declined, but then a sustained Que blood concentration was observed until 24 h post-injection, with mean residence times significantly longer than those of free Que [172].

Polyelectrolyte complex (PEC) NPs obtained through supramolecular self-organizing interaction between negatively charged lecithin and positively charged Cs were prepared by a nanoprecipitation method and evaluated for skin delivery of Que [173]. The obtained NPs presented a diameter of 95.3 nm with Que encapsulation efficiency and loading values ranging from 10.0 to 51.1% and from 0.5 to 2.5%, respectively. NPs resulted in enhanced ex vivo cumulative amounts in the dermis and epidermis within 12 h of application in comparison with Que-propylene glycol solution [173]. Similarly, in vivo penetration behavior investigation demonstrated that the particles could promote the accumulation of Que in the epidermis. Moreover, the skin treated with the NP suspension appeared to have an increased overall thickness, with a swollen stratum corneum in which loose cell junctions and increased intercellular space are evident. The obtained NPs formulation resulted in being a promising vehicle for the topical delivery of Que, increasing the permeation of the drug into the skin.

Recently, a microfluidic system was employed for the development of Que nanocrystals [174]. This technique is based on the precipitation of drug particles from a solution by the addition of a non-solvent. One of the advantages of microfluidic devices is the limited mixing path which allows the processing of small volumes of liquid under predictable and reproducible mixing conditions. In particular, by employing a centrifugal flow reactor, different processing parameters were investigated, including the type (Kolliphor P 407, Kolliphor P 188, Polysorbate 20) and the concentration (1–4%) of the stabilizer, reactor configuration (spiral-shaped- or straight-shaped-mixing channel), centrifuge rotation speed, and solution viscosity. Nanocrystals with a therapeutically relevant diameter (190–302 nm) were obtained, confirming the suitability of this innovative technique for the production of flavonoid nanocrystals [174].

Que-loaded hyaluronic acid (HA) nanohydrogels with an average size of 197.3 nm and a Que loading of 0.4 mmol/g were prepared by solvent non-solvent method [175]. The developed formulations were studied for the treatment of glioblastoma multiforme. Que’s anticancer, antioxidant and anti-inflammatory activities resulted in being higher for encapsulated Que than for free drug when tested in vitro using A172 and T98MG human glioblastoma cancer cells.

NMs for the delivery of Que based on Pluronic, possibly in combination with TPGS, were prepared employing a thin film evaporation method [176]. Que-loaded Pluronic-based NMs size ranged from 8.9 to 29.0 nm depending on Que loading and polymer composition. Que encapsulation efficiency and loading ranged from 38.0 to 89.0% and 5.8 to 10.6%, respectively, depending on the molar ratio of Pluronic to TPGS. Que in vitro release profiles confirmed the sustained release properties of the NMs showing that after 60 h 20–30% of the initially encapsulated drug was still entrapped in the NMs. In vitro cytotoxicity studies revealed that Que-loaded NMs presented a higher cytotoxic effect against MCF-7 breast cancer cells and MCF-7/ADR multidrug-resistant breast cancer cells in comparison with free Que [176].

Mixed NMs prepared by a thin film hydration method and based on the combination of different surfactants (Pluronic P123, L92, and P407, with and without TPGS) were loaded with Que to evaluate their activity against breast, ovarian, and multidrug-resistant cancer cells [177]. NMs size ranged from 18.7 to 403.2 nm. Que encapsulation efficiency and loading ranged from 14.7 to 94.8% and from 4.0 to 13.0%, respectively. Two optimized NMs formulations were characterized in vitro, showing a sustained release of Que that was significantly faster in the case of higher TPGS concentration as a consequence of increased hydrophilicity. Both optimized NMs exhibited higher total antioxidant capacity and cytotoxicity against SKOV-3 and MDA-MB-231 cells in comparison with free Que [177]. 

**Figure 19:**
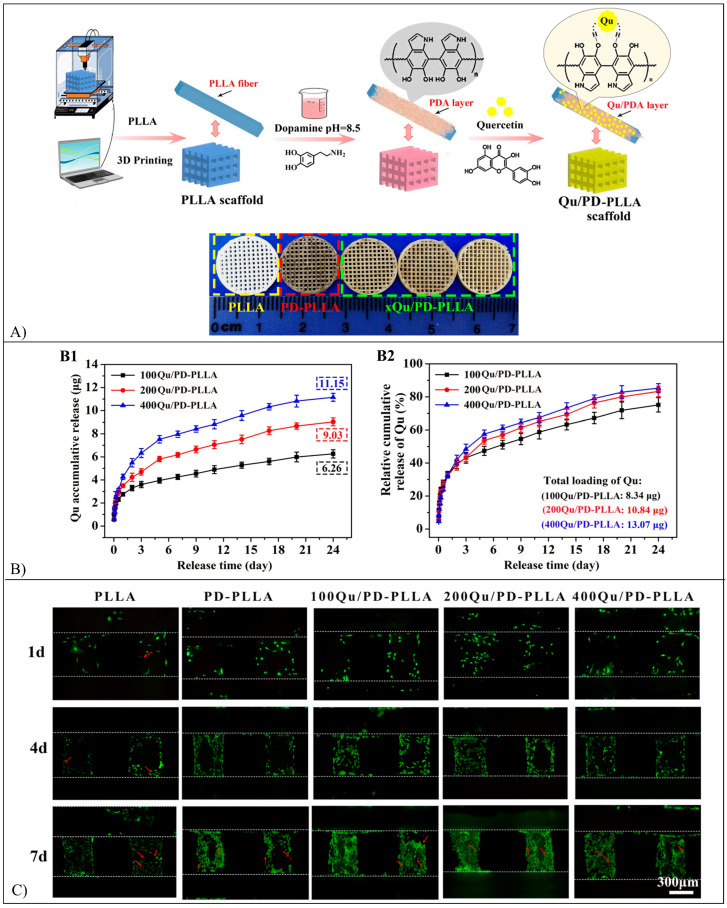
(**A**) Schematic representation of the scaffolds fabrication and functionalization procedure and representative images of the obtained scaffolds: PLLA scaffold, PDA-functionalized PLLA scaffold (PD-PLLA), and PD-PLLA scaffolds loaded with different amounts of Que (xQu/PD-PLLA); (**B**) (**B1**) Cumulative release quantity and (**B2**) the relative cumulative release of Que with time, and (**C**) Images of live/dead fluorescent staining of MC3T3-E1 cells on the PLLA, PD-PLLA, and Qu/PD-PLLA scaffolds after 1, 4, and 7 days of culture. Live cells are green stained, while dead cells are red stained and indicated with arrows. Reproduced with permission from [14], ACS Publications, 2019.

Que was also immobilized on the surface of 3D-printed PLLA through a polydopamine (PDA) intermediate layer formed by the oxidative self-polymerization of dopamine (DOPA) [14]. The PLLA scaffolds were printed with a square pore size of about 450 µm (Figure 19A). Different amounts of Que were immobilized on the devices (8.3, 10.8, and 13.1 µg). The devices were characterized by an in vitro Que release profile constituted by an initial burst release phase (almost 3 µg of Que released within the first 12 h), followed by a sustained release phase (Figure 19B). After 24 days, 75.1, 83.3, and 85.3% of immobilized Que were released, respectively, from the surface of the scaffolds at increasing loading concentration. Que-loaded PLLA scaffolds were able to promote preosteoblastic cells (MC3T3-E1) attachment and proliferation (Figure 19C), as well as to upregulate ALP activity and calcium nodules and to promote the expression of osteogenic-related genes and proteins [14].

Thermosensitive hydrogel scaffolds constituted by Cs and Col were loaded with Que to increase their osteoconductive and antioxidant properties [13]. The hydrogel scaffolds fabricated through sol-gel transition had a median pore size of 69.8 and 125.9 µm and a porosity of 89.4 and 66.7%, depending on Cs/Col ratio, 2:1 and 1:1, respectively. The scaffolds gradually released Que in vitro, showing a maximum cumulative release after 48 h of incubation and a sustained release up to 7 days. Scaffolds with a higher amount of Col showed faster Que release. Que-loaded Cs/Col 2:1 scaffolds demonstrated antioxidant properties and supported human periodontal ligament stem cells (hPDLSCs) viability and growth [13].

### 5.14. Rutin

Rutin (Rut): (3-((6-O-(6-Deoxy-alpha-L-mannopyranosyl)-beta-D-glucopyranosyl)oxy)-2-(3,4-dihydroxyphenyl)-5,7-dihydroxy-4H-1-benzopyran-4-one) (Figure 8) found in many plants, and buckwheat (*Fagopyrum esculentum Moench*) is reported as a major source of natural rutin [178]. It exerts many biological activities, such as anticancer, antioxidant, anti-bacterial, and anti-inflammatory activity [178]. 

The application of nanotechnology coupled with surfactant coating is a strategy proposed to enhance Rut transport across the blood-brain barrier (BBB) and exploit its anti-Alzheimer disease effect. The most used surfactants are Tween^®^ 80, TPGS, and polyethylene glycol-15-hydroxy stearate (Solutol^®^ HS 15). The targeting of Rut to the brain was obtained through lipid/polymer hybrid NPs (LPHNPs), combining the compatibility, degradability, and tendency to cross BBB naturally of the lipid nanocarriers with the stability of the polymeric matrix [179]. The system was prepared through a nanoprecipitation method employing PLGA as the polymer fraction and soybean phosphatidylcholine (SPC, Lipoid^®^ 100) as the lipid fraction. Different surface active agents (SAA) were employed, in particular, Tween^®^ 80, TPGS, and Solutol^®^ Hs 15. An in vitro release study demonstrated a gradual drug release over 6 h for all particle formulations. The pharmacokinetics of Rut-loaded NPs was explored in rats by tail vein injection. A significant improvement in Rut bioavailability was reached for all the formulations, likely thanks to the stealth effect triggered by the PEG moieties in SAA structures. Moreover, Tween and Solutol were found to increase the systemic availability of Rut, leading to comparable brain cell uptake [179].

NPs made of PLGA or Zein were loaded with Rut to investigate their antioxidant ability in comparison with free drugs [180]. The NPs were obtained through nanoprecipitation with a resulting average diameter of around 130 nm. The maximum Rut encapsulation efficiency, achieved using an anionic surfactant, was 17% for PLGA and 88% for Zein. In vitro Rut release rate from Zein NPs was fairly constant over time, and an increase in Rut concentration determined a decrease in its release rate, confirming the occurrence of significant flavonoid-protein interactions. Investigation on Rut release from PLGA NPs confirmed the poor ability of the polyester matrix to retain the flavonoid since a great amount of the active compound was released within a few hours. When tested in vitro using human chondrocytes (C-28 cell line) and keratinocytes (NCTC2544 cell line), Rut-Zein NPs exerted an enhanced antioxidant effect in comparison with free drugs. This protective ability can be related to the significant cell uptake of the colloidal system, as well as the synergistic action between the intrinsic antioxidant activity of the protein, which contains some xanthophyll pigments and aminoacids that can act as radical scavengers, and the pharmacological activity of the drug.

Polysaccharides were also evaluated for the encapsulation of Rut and its protection against degradation in the gastric environment. Rut and its aglycone Que were first encapsulated into cellulose acetate phthalate (CAP) and cellulose acetate trimellitate (CAT) microparticles obtained by spray-drying [181]. Depending on the polymer-to-drug ratio and formulation solvent, particle size ranged from 70.7 to 180.0 µm for Que-loaded particles and from 81.7 to 185.7 µm for Rut-loaded particles. Que and Rut loading values ended up being similar to the theoretical values ranging from 10.2 to 48.0% and from 14.6 to 49.0%, respectively. This was because encapsulation efficiency values ranged from 64.0 to 96.4% and from 87.0 to 98.0%, respectively. The evaluation of in vitro release kinetics at pH 1.0 confirmed that these particles were able to prevent Que and Rut exposure to an acidic medium, as well as to release the encapsulated drug when increasing the pH to 6.8 with a superimposable profile to that of the free drug [181]. Cs NPs prepared using the ionic gelation method were also studied to increase Rut stability in the gastric environment [182]. Particles’ mean diameter ranged from 322 to 814 nm by decreasing the polymer-to-TPP ratio, while Rut encapsulation efficiency increased from 3.7% to 57.6%. No Rut release occurred at pH 1.4, assuring the stability of the encapsulated drug in the gastric environment. When immersed in SIF at pH 7.7, the particles presented an initial 20% cumulative Rut burst release over the first 30 min, followed by no further release for the duration of the experiment (4 h) [181]. 

### 5.15. Xanthohumol

Xanthohumol (Xan): (1-(2,4-Dihydroxy-6-methoxy-3-(3-methylbut-2-en-1-yl)phenyl)-3-(4-hydroxyphenyl)prop-2-en-1-one) (Figure 7) is a prenylated chalcone flavonoid found in plants such as *Humulus lupulus* L. [183] with a wide spectrum of biological activities, such has anticancer [184,185,186], anti-inflammatory [187], antioxidant [188], anti-obesity [189], and antimicrobial [190] activity, as well as a protective effect on the liver [191].

**Figure 20:**
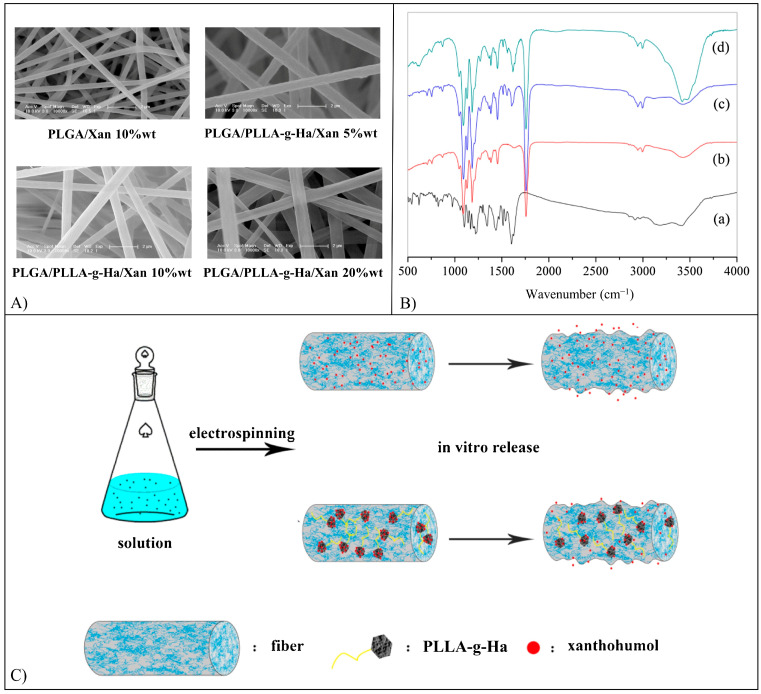
(**A**) SEM micrographs of binary PLGA meshes (MHs) (PLGA/Xan) loaded with 10%wt. of Xan and ternary PLGA MHs (PLGA/PLLA-g-Ha/Xan) loaded with 5%wt. of PLLA-g-Ha NPs and 5, 10, or 20%wt. of Xan; (**B**) FT-IR spectra of (a) pure Xan, (b) PLGA mesh, (c) binary mesh (PLGA/Xan 10%wt.), (d) ternary mesh (PLGA/PLLA-g-HA/Xan 10%wt.), and (**C**) schematic model describing Xan release from binary (PLGA/Xan) and ternary mesh (PLGA/PLLA-g-Ha/Xan). Reproduced with permission from [192], Elsevier, 2016.

Xan-loaded meshes (MHs) constituted by PLGA, and PLLA-grafted nanohydroxyapatite (PLLA-g-Ha) were fabricated employing binary-solvent (chloroform:N,N-dimethylformamide 85:15 *v/v*) electrospinning [192]. The fabricated MHs showed a well-defined fiber texture with a coarse fiber surface due to the addition of PLLA-g-Ha (Figure 20A). The average fiber diameter increased from 680 to 950 nm when increasing Xan content from 5 to 20%wt. FT-IR analysis (Figure 20B) showed for binary MHs (constituted only by PLGA and Xan) a reduction of the absorbance of the band at 3400 cm^−1^, characteristic of the -OH group stretching, in comparison with that of the pure drug, likely because of breaking of the hydrogen bonds between drug molecules due to the dispersion in the polymer matrix. The same analysis carried out on the ternary mesh (constituted by PLGA, Xan, and PLLA-g-Ha) instead showed an increase in the absorbance of this band, which may be related to the formation of hydrogen bonds between residual -OH groups of Ha and -OH groups of Xan. In vitro, Xan release investigation demonstrated that the samples were characterized by a burst release phase within the first 4 h followed by a sustained release phase, whose rate could be increased by increasing the content of loaded Xan. By comparing the Xan release profile of MHs loaded with 10%wt. of Xan with and without the presence of PLLA-g-Ha, it was evident that the binary MHs showed a faster Xan release, likely because of hydrogen bonds between drug molecules and PLLA-g-Ha in ternary MHs (Figure 20C). In vitro cell culture test revealed that the ternary MHs supported MC3T3 preosteoblast cells viability at a level comparable to those of blank PLGA and Xan-loaded PLGA MHs [192].

**Table 1:** Drug delivery systems (DDS) for flavonoid encapsulation and controlled release, relevant employed polymers and fabrication techniques, and most important, in vitro and in vivo results.

Flavonoid	DDS ^a^	Polymer (s)	Fabrication Technique (s)	In Vitro and In Vivo Results	Refs.
Baicalin	NCs	PLGA	Nanoprecipitation	Apoptotic effect on MCF-7, MDA-MB-231 and A549 cells; reduction of inflammatory markers in rats and in newborn skin pig.	[68,69,70,71,72]
	Nanogels	Gell-cholesterol	Ionotropic gelation		
	NMs	Pluronic F68-SA	Thin film hydration		
	Transfersomes	Gell-cholesterol	Thin film hydration		
Cirsiliol	NCs	PEG-PCL	Nanoprecipitation	Antiproliferative effect against MCF-7 cells.	[74]
Chrysin	NCs	PLGA	Nanoprecipitation	Reduction of serum blood glucose level in rats; reduction of growth of HGCCs ^c^ cells and of miR expression; anti-growth effect on T47D, MCF-7, Caco-2, HT29 cells. Repolarization of peritoneal macrophages to anti-inflammatory phenotype. Correction of drug-induced neurodegeneration. Increased viability of hADSCs ^b^ and expression of stemness genes, increased DPSCs ^d^ viability and proliferation and differentiation towards osteoblasts of MSCs ^e^.	[84,85,86,87,88,89,90,91,92,93,94,95]
	NPs	PLGA-PEG, PLGA-PEG-PLGA, BSA	Single/double emulsion solvent evaporation, nanoprecipitation		
	Nanovesicles	Cs	Ethanol injection		
	MHs	PCL-PEG-PCL	Electrospinning		
	SCs	PCL-Gel, Cmc/Cs/HA	Freeze drying, ionic gelation		
Fisetin	NMs	PEG-PCL, PLA-TPGS	Self-assembly, nanoprecipitation	Inhibition of subcutaneous tumor growth, MCF-7 cell proliferation, and α-glucosidase activity. Antitumor effect against HCT116 cells, reduction of the tumor growth in rats.	[101,102,103,104]
	NPs	PCL/PLGA-PEG-COOH, PLA	Single emulsion solvent evaporation		
Icariin	NMs	PEG-PLGA	Nanoprecipitation	Cytotoxic effect against ASPC-1 cells. Increased proliferation of MG-63 and MC3T3-E1 cells, increased hADSCs and BMMSCs ^f^ differentiation, increased regeneration of bone in rats and in rabbits	[110,111,112,113,114,115,116,117,118,119,120,121,122]
	SCs	PHBV, PLCL/SF, PCL/Col-Cs/Col, PLGA/, SIS, Cs/GP, ADA/Gel/MSN, Col, HA-MA/Col	Solvent casting salt leaching, coaxial electrospinning, freeze drying, low-temperature 3D printing, sol-gel transition, 3D printing, Solvent casting UV irradiation		
	MPs	PLGA	Single emulsion solvent evaporation		
Icaritin	NMs	Soluplus^®^/Poloxamer 407	ABS	Anti-HCC effect. Increased growth of BMMSCs and their differentiation into osteoblasts, healing of bone defects in rabbits and in adult emu.	[126,127,128,129,130]
	NPs	PLGA-PEG	Nanoprecipitation		
	SCs	PLGA	Low-temperature 3D printing		
Morin	NPs	HA-PBCA/TPGS	Dialysis	Cytotoxic effect against A549 cells. Antibacterial activity against *A. naeslundii* and *S. mutans*.	[134,135]
	MPs	Alg/Gell	Ionotropic gelation		
	Films	Alg/Gell	Solvent casting		
	Tablets	Alg/Gell	Freeze drying		
Myricetin	NPs	PDMAEMA-b-poly-(dimethylaminoethyl methacrylate-co-butyl methacrylate-co-propylacrylic acid)/PDMAEMA	Self-assembly	Antimicrobial and anti-biofilm properties; enhanced brain accumulation and penetration efficiency.	[136,137,138]
	NCs	Eudragit RS100^®^	Nanoprecipitation		
	NMs	Cs-Pluronic P123/F68	Thin film hydration		
Naringenin	NPs	PLGA, PLA/PVA, zein/pectin, Eudragit E100^®^, Cs/Alg, Cs	Nanoprecipitation, single emulsion solvent evaporation, ionotropic gelation	Increased brain accumulation, diabetogenic effects in rats, suppression of colorectal cancer growth.	[141,142,143,144,145,146,147]
	NXs	PVP	Thin film hydration		
Naringin	MPs	CAP	Spray drying	Reduction of chronic arthritis in rats.	[150,151]
	NPs	PLGA	Single emulsion solvent evaporation		
Oroxylin A	PDots	Conjugated polymer	Nanoprecipitation	Cytotoxic effect against A431 cells and preferential targeting of tumor tissue.	[153]
Phloretin	NPs	Cs	Ionic gelation	Cytotoxic effect against KB and SKMEL cells.	[156,157]
	NCs	PCL	Nanoprecipitation		
Quercetin	NPs	PLGA, PCL, PCL-TPGS, BSA, BSA/PPG-PEG-PPG, Cs, NCS/Alg, CMCA, CMCA-GA, lecithin/Cs, HA	Nanoprecipitation, electrospraying, single/double emulsion solvent evaporation, ionic gelation, dialysis, microfluidic	Cytotoxic activity against A549, SKBR3, HepG2, A172, T98MG, MCF-7, MCF-7/ADR, SKOV-3, MDA-MB-231 cells. Increase of diuretic action in rats, adhesion and proliferation of H9c2 heart cells. Reduction of Alzheimer’s disease malignancy, sodium oxalate-induced cytotoxicity on Maldin-Darby canine kidney epithelial cells. Increase of proliferation and differentiation of MC3T3-E1 cells and proliferation of hPDLSCs ^g^	[13,14,159,160,161,162,163,164,165,166,167,168,169,170,171,172,173,174,175,176,177]
	NMs	Pluronic-TPGS, Pluronic P123/L92/P407	Thin film evaporation		
	SCs	PLLA, Cs/Col	Fused deposition modeling, sol-gel transitions		
Rutin	MPs	CAP, CAT	Spray drying	Antioxidant effect on C-28 and NCTC2544 cells	[179,180,181]
	NPs	PLGA, zein, Cs	Nanoprecipitation, ionotropic gelation		
Xanthohumol	MHs	PLGA	Electrospinning	Ability to support MC3T3-E1 cells viability	[192]

^a^ drug delivery systems (DDS): nanocapsules (NCs), nanoparticles (NPs), microparticles (MPs), nanomicelles (NMs), meshes (MHs), scaffolds (SCs); ^b^ human adipose-derived stem cells (hADSCs); ^c^ human gastric carcinoma cells (HGCCs); ^d^ dental pulp stem cells (DPSCs); ^e^ mesenchymal stem cells (MSCs); ^f^ bone marrow mesenchymal stem cells (BMMSCs); ^g^ human periodontal ligament stem cells (hPDLSCs).

## 6. Conclusions and Future Perspectives

Flavonoids are natural molecules present in foods and beverages and have been employed in traditional Chinese medicine since ancient times. They present a wide range of beneficial properties, e.g., antioxidant, anti-inflammatory, anticancer, antidiabetic, antibacterial, antiviral, hepatoprotective, cardioprotective, and neuroprotective activity. To overcome their low bioavailability, a great variety of flavonoids have been encapsulated into many different polymeric devices. Among others, Chr, Ica, Nag, and Que are the most investigated flavonoids in this context. Materials and formulation strategies have been tailored to the physical-chemical properties of the investigated flavonoid in order to optimize drug loading and release kinetics, as well as morphological and dimensional features of the targeted polymeric system. This approach allows increased control over the release rate, time, and place of flavonoids, in order to optimize their biological activity. In vivo studies carried out on mice, rabbits and even large animal models confirmed the enhanced bioavailability and bioactivity of flavonoids after encapsulation into polymeric devices, in comparison to those achieved with injected or orally taken free compounds. Future research should focus on the clinical trial investigation of optimized flavonoid formulations to propel their translation into the biomedical market [193].

## Data Availability

Not applicable.

## Abbreviations

2D	Two dimensional
3D	Three dimensional
5-Fu	5-fluorouracil
ABS	Acid-base shift
ABTS•+	3-ethylbezothiazoline-6-sulfonic acid
ADA	Alginate dialdehyde
Alg	Alginate
ALP	Alkaline lysis phosphatase
ASCs/ADSCs	Adipose-derived stem cells
Aβ	Amiloid-β peptide
Bai	Baicalin
BBB	Blood-brain barrier
BMSCs/BMMSCs	Bone marrow mesenchymal stem cells
BSA	Bovine serum albumin
CAP	Cellulose acetate phthalate
CAT	Cellulose acetate trimellitate
Chr	Chrysin
Cir	Cirsiliol
CLSM	Confocal laser scanning microscopy
Cmc	Carboxymethylcellulose
CMCA	O-carboxymethylated chitosan modified with cholic acid
Col	Collagen
Cs	Chitosan
Cur	Curcumin
DCF	Dichlorofluorescein
DDS	Drug delivery system
DMAEMA	2-(dimethylamino)ethyl methacrylate
DMSO	Dimethyl sulfoxide
dMyr	Dihydromyricetin
DOPA	Dopamine
Dox	Doxorubicin
DPSCs	Dental pulp stem cells
ECM	Extracellular matrix
EMA	Ethyl methacrylate
FA	Folic acid
FDA	Food and drug administration
Fis	Fisetin
FT-IR	Fourier transformed infra red
GA	Glycirrhetinic acid
Gel	Gelatin
Gell	Gellan gum
Gly	Glycine
GP	Glycerophosphate
HA	Hyaluronic acid
Ha	Hydroxyapatite
HA-MA	Hyaluronic acid methacrylate
HAS	Human serum albumin
HGCCs	Human gastric carcinoma cells
HNT	Halloysite nanotubes
HPLC	High performance liquid chromatography
Hyp	Hydroxyproline
IC50	Half maximal inhibitory concentration
Ica	Icariin
Ica-MA	Icariin methacrylate
Ict	Icaritin
IL-1β	Interleukin-1β
LPHNPs	Lipid/polymer hybrid NPs
MAA	Methacrylic acid
MH	Morin hydrate
MHs	Meshes
MI	Icariin-loaded microparticles
miR	Micro-ribonucleic acid
MMA	Methyl methacrylate
mNPs	Mixed polymers-based nanoparticles
Mor	Morin
MPs	Microparticles
MSCs	Mesenchymal stem cells
MSN	SiO2-CaO mesoporous nanoparticles
Myr	Myricetin
Nag	Naringenin
Nar	Naringin
NCs	Nanocapsules
NCS	N-succinyl chitosan
NMs	Nanomicelles
NPs	Nanoparticles
NXs	Nanocomplexes
OCMC	O-carboxymethylated chitosan
OrA	Oroxylin A
P/P NPs	Poly(lactic acid)/poly(vinyl alcohol) nanoparticles
P4HB	Poly(4-hydroxybutyrate)
PAA	Poly(acrylic acid)
PBCA	Poly(buthyl cyano acrylate)
PBS	Phosphate buffer saline
PCL	Poly(ε-caprolactone)
PDA	Poly(dopamine)
PDMAEMA	Poly(2-(dimethylamino)ethyl methacrylate)
Pec	Pectin
PEC	Polyelectrolite complex
PEG	Poly(ethylene glycol)
PEO	Poly(ethylene oxide)
PGA	Poly(glycolic acid)
P-gp	P-glycoprotein
PHA	Polyhydroxyalkanoates
PHB	Poly(3-hydroxybutyrate)
PHBHHx	Poly(3-hydroxybutyrate-co-3-hydroxyexanoate)
PHBV	Poly(3-hydroxybuthyrate-co-3-hydroxyvalerate)
Phl	Phloretin
PLA	Poly(lactic acid)
PLCL	Poly(lactic acid-co-ε-caprolactone)
PLGA	Poly(lactico-co-glycolic acid)
PLLA	Poly(L-lactic acid)
PMAA	Poly(methacrylic acid)
PMI	Poly(lactic-co-glycolic acid) microspheres containing icariin-loaded microparticles
PMI-H	PMI loaded with high amount of icariin
PMI-L	PMI loaded with low amount of icariin
PMI-M	PMI loaded with medium amount of icariin
PPO	Poly(propylene oxide)
Pro	Proline
PVA	Poly(vinyl alcohol)
PVP	Poly(vinyl pyrrolidone)
Que	Quercetin
ROS	Reactive oxygen species
Rut	Rutin
SA	Stearic acid
SAA	Surface active agent
SAON	Steroid associated osteonecrosis
SCd	Sulfobuthylether-β-cyclodextrin
SCs	Scaffolds
SEM	Scanning electron microscopy
SF	Silk fibroin
SGF	Simulated gastric fluid
SIF	Simulated intestinal fluid
SiN	Silica nanoparticles
SIS	Small intestine submucosa
SPC	Soybean phosphatidylcholine
TCP	Tricalcium phosphate
TEM	Transmission electron microscopy
THF	Tetrahydrofuran
TNF-α	Tumor necrosis factor-α
TPA	12-O-tetradecanoylphorbol-13-acetate
TPGS	D-α-tocopheryl polyethylene glycol 1000 succinate
TPP	Sodium tripolyphosphate
UV	Ultraviolet
Xan	Xanthohumol
Z/P NPs	Zein/pectin nanoparticles
ε-CL	Ε-caprolactone
µ-CT	Micro-computed tomography
